# DriveWays: a method for identifying possibly overlapping driver pathways in cancer

**DOI:** 10.1038/s41598-020-78852-8

**Published:** 2020-12-15

**Authors:** Ilyes Baali, Cesim Erten, Hilal Kazan

**Affiliations:** 1 Electrical and Computer Engineering Graduate Program, Antalya Bilim University, 07190 Antalya, Turkey; 2 Department of Computer Engineering, Antalya Bilim University, 07190 Antalya, Turkey

**Keywords:** Cancer genomics, Computational biology and bioinformatics, Systems biology

## Abstract

The majority of the previous methods for identifying cancer driver modules output nonoverlapping modules. This assumption is biologically inaccurate as genes can participate in multiple molecular pathways. This is particularly true for cancer-associated genes as many of them are network hubs connecting functionally distinct set of genes. It is important to provide combinatorial optimization problem definitions modeling this biological phenomenon and to suggest efficient algorithms for its solution. We provide a formal definition of the Overlapping Driver Module Identification in Cancer (ODMIC) problem. We show that the problem is NP-hard. We propose a seed-and-extend based heuristic named DriveWays that identifies overlapping cancer driver modules from the graph built from the IntAct PPI network. DriveWays incorporates mutual exclusivity, coverage, and the network connectivity information of the genes. We show that DriveWays outperforms the state-of-the-art methods in recovering well-known cancer driver genes performed on TCGA pan-cancer data. Additionally, DriveWay’s output modules show a stronger enrichment for the reference pathways in almost all cases. Overall, we show that enabling modules to overlap improves the recovery of functional pathways filtered with known cancer drivers, which essentially constitute the reference set of cancer-related pathways.

## Introduction

Recent advances in high-throughput DNA sequencing technology have allowed several projects such as The Cancer Genome Atlas (TCGA) to systematically generate genomic data for thousands of tumors across many cancer types^1^. A key fundamental challenge in cancer genomics is to distinguish functional mutations that drive tumorigenesis, or *drivers*, from the numerous *passenger* mutations that occur randomly but that are not directly associated with cancer development. Several methods to identify and rank cancer driver genes have been developed^2–9^; see^10,11^ for comprehensive evaluations and surveys on the topic.

Such a challenge is further complicated by the highly interactive nature of genes/proteins, thus necessitating the identification not only of such drivers but also of modules consisting of webs of drivers culpable in cancer initation and progression. Several computational approaches have been proposed for the cancer driver module identification problem and they can be categorized according to the types of biological data they utilize and the proposed optimization functions to model the underlying biological problem. Early approaches for driver module analysis have primarily utilized the mutation data, in particular the frequency of mutations^12–14^, the positional clustering of mutations^15^, or the co-occurence of mutations in the same patients^16^. These methods can provide limited results as cancer genomes exhibit extensive mutational heterogeneity. Multiple approaches have been proposed to alleviate this problem. Rather than using mutation frequencies directly, Hotnet2 applies a random walk strategy to diffuse the mutation frequencies throughout the network and then identifies driver modules as strongly connected components of the resulting network^17^. TieDIE is also based on a diffusion strategy but different from Hotnet2 it uses the network diffusion approach to connect genomic perturbations to gene expression changes characteristic of cancer subtypes. Another direction is to utilize the concept of mutual exclusivity, the fact that multiple alterations in the same functional pathway occur less frequently because of diminished selective pressure. There exist methods that calculate all pairwise mutual exclusion scores^18,19^. However, most methods limit the search space by using prior interaction knowledge. For instance, Ciriello *et al.* test each clique in the interaction network against random permutations to estimate the significance of mutation overlaps^20^. Vandin *et al.* propose a score that rewards coverage and penalizes mutation overlaps, and then searches for a set of genes that maximizes this score^21^. The same scoring function is also utilized by follow-up methods with an extension on the search technique^22,23^. Babur *et al.* improves over the scoring function of^21^ by fixing the bias towards highly altered genes^24^. Their proposed statistical mutual exclusivity test is used within a greedy search to identify groups of genes with high mutual exclusivity. MEMCover is also based on a greedy iterative seed-and-extend heuristic where a function that integrates coverage, mutual exclusivity and confidence values of interactions in the network is maximized^25^. MEXCOWalk extends Hotnet2’s random walk strategy by introducing edge weights that include mutual exclusity and coverage^26^.

A common theme in almost all the mentioned cancer module identification methods is to search for nonoverlapping modules. However, biological pathways often overlap since proteins may carry out more than one function or belong to more than one protein complex^27^. Protein multifunctionality can also be considered as a means to coordinate multiple cellular activities serving as switches between pathways. As such, current methods that ultimately aim to provide a subset of existing biological pathways that are associated with cancer assume a problem definition that does not reflect the nature of biological pathways. To the best of our knowledge, only two previous methods provide possibly overlapping cancer driver modules, MEMCover^25^ and ModulOmics^28^. In the former, no criteria for overlaps is included in the main search procedure which produces nonoverlapping output modules. The possible overlaps are only achieved via an optional post-processing step and no performance evaluations are done for this setting. ModulOmics integrates PPI network proximity, mutual exclusivity of DNA alterations, and RNA level coregulation and coexpression, into a single probabilistic framework, by simultaneously optimizing over all four model components. A significant shortcoming of ModulOmics is the lack of control over the amount of overlaps between the driver modules. For instance, for breast cancer, ModulOmics provides its top 50 modules, ranging in size 2–4, in the published results. Among these top 50 modules many of them are almost the same; 402 pairs differ only by one gene.

On the other hand there are some related problems in a wide range of areas including biological networks and social networks, such as protein complex identification or community detection, where overlapping module identification is an important research topic; see^29^ for a survey on the topic. Shih *et al.* propose a soft variation of regularized Markov clustering to enable the identification of overlapping clusters in PPI networks^30^. ClusterOne uses a modularity metric in a weighted graph to guide the search for finding possibly overlapping subgraphs that correspond to protein complexes^31^. Bennett *et al.* propose a mixed integer nonlinear programming model to transform non-overlapping modules to overlapping, and apply this method to PPI networks of multiple organisms^32^. Although the proposed methods provide valuable insight on overlapping module constructions in the general setting, they are not designed for finding disease-associated modules. Modeling disease association requires extensive changes both in the input data and on the search procedure.

We propose DriveWays designed to identify potentially overlapping cancer driver modules. DriveWays uses a seed-and-extend strategy on a PPI network where it adds or removes gene sets based on a novel scoring function that includes coverage and mutual exclusivity of the module. The sizes of output modules can be controlled via appropriate parameters. We show that DriveWays improves over existing methods in the recovery of known cancer genes and more importantly in the recovery of pathways of known cancer driver genes. For the latter, we propose novel evaluation strategies that should prove useful for further research in this area.

## Methods

Given the mutations data from a cancer cohort and a H. Sapiens PPI network, the informal goal is to extract from the PPI network subsets of genes (modules) that best reflect pathways related to the cancer under study. Ideally, these should correspond to the important causal functional pathways of driver genes of the relevant cancer. We first provide a computational problem definition to model this biological phenomenon. We discuss the computational complexity of the problem and provide an efficient greedy heuristic algorithm.

### Problem definition

Let $G=(V,E)$ represent the PPI network where each vertex $u_i\in V$ denotes a gene $g_i$ whose expression gives rise to the corresponding protein in the network and each undirected edge $(u_i,u_j)\in E$ denotes the interaction among the proteins corresponding to genes $g_i,g_j$. Henceforth assume $g_i$ denotes both the gene and the corresponding vertex $u_i$ in *G*. Let $S_i$ denote the set of samples for which $g_i$ is mutated and *S* denote the list of all such sets. Let $M\subseteq V$ be a set of genes denoting a *module*. Let *G*(*M*) denote the subgraph of *G* induced by the vertices corresponding to genes in *M*.

Since a driver pathway tends to be perturbed in a relatively large number of patients, one of the desired properties of each module is large *coverage*^17,25,26^. We define the coverage of *M* as, $COV(M) =\frac{\left|\bigcup _{\forall g_i\in M}S_i\right|}{\left|\bigcup _{\forall g_j\in V} S_j\right|}$. Several cancer driver module identification methods have additionally made use of the concept of *mutual exclusivity*^20,24–26,33^. It refers to the phenomenon that for a group of genes which exhibit evidence of shared functional pathway, simultaneous mutations in the same patients are less frequent than is expected by chance^18^. Formally, we define the mutual exclusivity of a module *M* as, $MEX(M) = \frac{\left|\bigcup _{\forall g_i\in M}S_i\right|}{\sum _{\forall g_i\in M}|S_i|}$. The functions *COV* and *MEX* are in many cases conflicting, in the sense that large coverage can be obtained at the expense of mutual exclusivity and vice versa. Therefore, similar to the module scoring function of Wu *et al.*^34^, we combine the two functions in a product form and define the *module score* of *M* as, $MS(M) = COV(M) \times MEX(M)$. An instance depicting the advantage of such a product form formula over an additive function can be found in the Supplementary Document, Section 1.1. Finally, for a set *D* of modules we define the *overlapping driver module set score* as, $ODMSS(D) = \sum _{\forall M\in D} MS(M)$.

Given as input a 4-tuple $\prec G, S, \delta _m, \delta _s\succ$, where $\delta _m$ and $\delta _s$ are integers, we define the *overlapping driver module identification in cancer (ODMIC)* problem as that of finding a set *D* of *possibly* overlapping modules that maximizes the *ODMSS*(*D*) and that satisfies the following:

- *Connectivity:* For each $M\in D$, *G*(*M*) is connected.
- *Uniqueness:* For each $M_i,M_j\in D$, $M_i\ne M_j$.
- *Minimum Size:* $$min_{\forall M\in D}|M|=\delta _m$$.
- *Total Size:* $$\sum _{\forall M\in D} |M|=\delta _s$$.

Note that the use of the phrase *overlapping* in the problem definition is not meant to imply a hard constraint on the existence of modules in *D* sharing some common genes; there may exist a solution set *D* consisting solely of pairwise disjoint modules. Such a choice of phrasing is due to the emphasis we want to place for the distinct property of our definition that allows the possibility of partial overlaps between modules in a solution set, unlike most of the existing driver module identification approaches which prohibitively place hard constraints to ensure all output modules are pairwise disjoint. Thus, henceforth we mean *possibly overlapping* whenever we employ the phrase *overlapping*.

The ODMIC problem definition is in part inspired by the cancer driver module identification problem definition of MEXCOWalk^26^. One crucial difference is that the MEXCOWalk definition does not allow overlaps. Secondly, due to the lack of overlaps, MEXCOWalk optimization score requires size normalizations in the contributions of MEX and COV. Furthermore the ODMIC scoring function is the sum of independent scores of modules and thus allows quite a different solution structure than that of MEXCOWalk. Finally, the size constraint of the output set of modules is with respect to the size of the set of unique genes in MEXCOWalk, whereas our problem definition applies the *Total Size* constraint which is determined by the sum of the sizes of the output modules. Such a choice allows flexible overlaps to be realized in an optimum solution. For instance, for $\delta _s=10$, a single module of size 10, two nonoverlapping modules of size 5 each, or two modules of size 5 with 4 common genes, all constitute legal instances in the solution space. Regardless of the differences in the problem definitions, we show that a reduction similar to the one employed in MEXCOWalk applies to this problem as well and that the problem in its generality is computationally intractable.

#### **Theorem 0.1**

*The ODMIC problem is NP-hard.*

#### *Proof*

See the Supplementary Information. $\square$

The following lemma provides further intuition on the ODMIC problem by stating a fact regarding the structure of an optimum solution.

#### **Lemma 0.2**

*There is an optimum solution* *D* *of the ODMIC problem on input instance* ${\prec}G, S, \delta _m, \delta _s{\succ}$, *where* $|M|<2\delta _m$, $\forall M\in D$.

#### *Proof*

See the Supplementary Document. $\square$

Due to this structural property the ODMIC problem admits a pseudo-polynomial time algorithm under a certain setting.

#### **Theorem 0.3**

*The ODMIC problem is solvable in pseudo-polynomial time for constant* $\delta _m$.

#### *Proof*

We propose a solution based on dynamic programming. Let *D* be an optimum solution of a given ODMIC input instance. By the *Minimum Size* constraint of the problem definition and by Lemma 0.2, we have $\delta _m\le |M|<2\delta _m$, for $M\in D$. Given a graph of *n* vertices, there are $O(n^{2\delta _m-1})$ induced connected subgraphs with the allowed sizes. Since $\delta _m$ is constant, in an enumeration $M_1, M_2\ldots , M_p$ of all such subgraphs we have $p=O(n^k)$, for constant *k*. Consider an optimum score table *c*, where *c*[*i*, *j*] indicates the optimum ODMSS score of an input instance consisting of subgraphs $M_1, M_2,\ldots M_i$ and the *Total Size* constraint set to *j*. Then $c[i,j]=max(c[i-1,j], c[i-1,j-|M_i|]+MS(M_i))$. Thus the optimum solution can be found in time $O(n^k\times \delta _s)$. $\square$

Although the above result is valuable in providing a theoretical intuition regarding the solution structure, it is not fit for many practical settings. Efficient algorithms that may be suboptimal but that provide solutions close to optimum by making careful design choices with respect to the optimization criteria of the ODMIC problem are necessary.

### DriveWays algorithm

We provide a polynomial-time heuristic algorithm, DriveWays, for the ODMIC problem. It is based on a greedy seed-and-extend procedure on the input PPI network, that incorporates mutual exclusivity and coverage information. The pseudocode is provided in Algorithm 1. There are two main steps of the algorithm: (1) rank the genes with respect to the *MS* scores within the immediate neighborhoods (2) initialize the module with the highest ranked seed and iteratively modify it by adding or removing a set of genes. The second step is repeated multiple times until we satisfy the ODMIC problem definition constraint regarding *Total Size*. Details are described in the following subsections.

#### Ranking the seeds

Prioritizing cancer genes based on a combined score of coverage and mutual exclusivity has been employed in several previous approaches^21,25,26^. In line with our ODMIC problem definition, we similarly make use of coverage and mutual exclusivity values in the form of our module score definition. We first filter out the genes that are mutated in less than 1% of the cohort. Then the remaining genes are sorted in nonincreasing order with respect to the module scores of their extended neighborhoods, that is, $MS(N_e(g))$, where $N_e(g)$ denotes the set of neighbors of gene *g* in *G*, together with *g*. Such a score is basically a measure of how fit a gene is for further immediate growth with the neighbors. We note that we assessed the importance of our seed ranking procedure by rerunning DriveWays with randomly selected seed lists. We observe that the modules obtained with randomly selected seeds perform significantly worse than our original set of output modules, in terms of all the evaluation criteria considered in this study; see Supplementary Figs. S3 and S4 for details. 
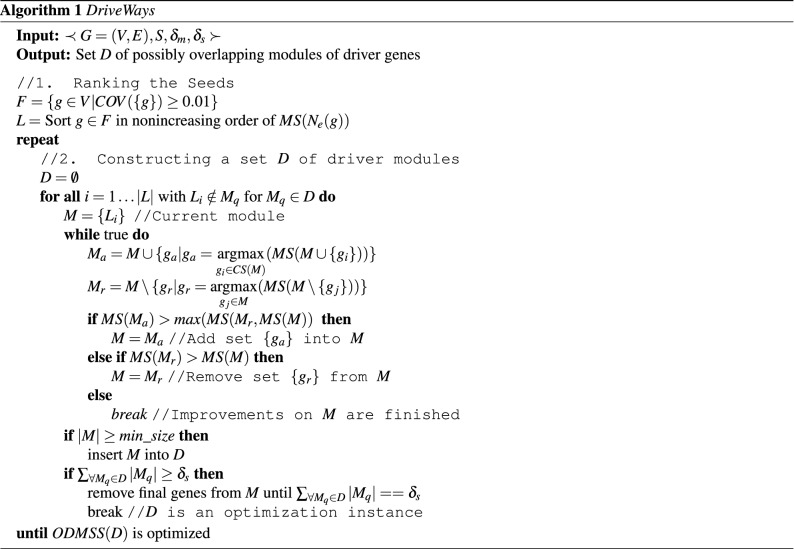


#### Constructing set of driver modules

We construct the set *D* of possibly overlapping modules through a greedy iterative module update procedure. For constructing a new module *M* to be added to *D*, we initialize *M* with the highest ranking seed that does not appear in an already existing module. We update *M* by either adding or removing certain gene(s) iteratively until such modifications no longer provide a gain to the current module in terms of the *MS* score. To check whether any gene additions to the current module provide a gain, we first construct a candidate set *CS*(*M*), from which the genes to be possibly added to *M* are selected. Let $N(g_i)$ denote the neighborhood of $g_i$ in *G* and let $N(M)=\bigcup _{\forall g_i\in M}N(g_i)$. A gene $g_i \in N(M)$ is added to *CS*(*M*), if it satisfies the following two conditions:

1 $$\begin{aligned}&\frac{ |\bigcup _{\forall g_k\in M \cup \{g_i\}}S_k| }{\sum _{g_k \in M } |S_k|} > t \end{aligned}$$

2 $$\begin{aligned}&\frac{deg(g_i, M)}{mean\_deg(g_i)} > 1 / d \end{aligned}$$

Inequality (1) relates the coverages of *M* with or without $g_i$ to the mutual exclusivity of *M*. More specifically, it requires that the new coverage of the module with $g_i$ should at least be a constant multiple *t* of the ratio of the old coverage to the old mutual exclusivity. In Inequality 2, $deg(g_i, M)$ denotes the degree of $g_i$ in the subgraph of *G* induced by $M\cup \{g_i\}$. On the other hand $mean\_deg(g_i)$ is the average across $deg(g_i,M_q)$ values, where $g_i\in M_q$, for already existing $M_q\in D$. Thus by Inequality (2) a gene $g_i$ is a candidate to be possibly added to the current module *M*, if it is well-connected to *M*, as compared to its connectivity to the already existing modules. Note that unlike the seed selection procedure, we do not impose any further constraints on the candidate set *CS*(*M*), other than the inequalities (1, 2). More specifically, a gene may be in the candidate set of the current module, even though it is a member of some of the previously constructed modules. This accounts for the *possibly overlapping* emphasis of the proposed approach, where a gene may simultaneously be a member of several output modules.

Let $M_a$ be the union of *M* with the set of genes $g_a\in CS(M)$ that maximize $MS(M\cup \{g_a\})$. Let $M_r$ be the difference of *M* with the set of genes $g_r\in M$ that maximize $MS(M\setminus \{g_r\})$. The modules $M_a,M_r$ compete in *MS* improvement; if at least one improves over *MS*(*M*), the one with larger improvement is committed on *M*. If no improvement is achieved, the modifications of *M* are finalized and it is added into *D*. This procedure of module updates from a single seed are continued until the sum of the sizes of the modules in *D* reaches $\delta _s$.

#### Optimizing with respect to parameters *t*, *d*

The parameter *t* in Inequality (1) indirectly controls the sizes of the output modules. Note that in the algorithm we do not explicitly control the module sizes in accordance with Lemma  0.2, since *t* achieves the same goal with more flexibility. The parameter *d* on the other hand, indirectly controls the amount of overlap between the modules. Setting *d* to a large value increases the likelihood of a gene satisfy Inequality (2) and thereby become a candidate for the current module, even if it is sparsely connected to the current module and is a member of many of the previously constructed modules. This in turn increases the amount of overlaps among the modules in the output set *D*. An important feature of the algorithm is to set the parameters *t*, *d* automatically via an optimization function which chooses the instance of *D* that maximizes the main optimization function, *ODMSS*(*D*), among several instances produced through different *t*, *d* settings. The *repeat* loop of the main algorithm corresponds to this procedure implemented with the Bayesian Optimization (BayesOpt) procedure of^35^.

## Results

We implemented the DriveWays algorithm in Python. The source code, useful scripts for evaluations, and all the input data are freely available as part of the Supplementary Information. We compare the results of DriveWays against those of four alternative methods. Among these alternatives three of them are knowledge-based cancer driver module identification methods: Hotnet2, MEMCover, and MEXCOwalk. As the fourth alternative method we employ ClusterOne and consider it as a baseline since it is a representative algorithm for community detection in networks and outputs overlapping modules without any reference to cancer-related data. MEMCover is chosen due to its close connection to our work. It also optimizes mutual exclusivity and coverage of modules with a greedy seed-and-extend heuristic. Moreover, MEMCover is able to provide overlapping modules via a post-processing step. Hotnet2 is a good representative of heat diffusion based module finding algorithms though it only considers coverage, whereas MEXCOwalk improves over Hotnet2 by introducing edge weights that consider both mutual exclusivity and coverage.

### Input data

All the methods except ClusterOne use the same input in the form of mutation data of available samples from TCGA and a PPI network. ClusterOne only uses the network information. We download the somatic aberration data from TCGA pan-cancer cohort preprocessed by^17^. Namely non-silent SNVs were extracted from Synapse (syn1710680), and GISTIC2 output CNAs were downloaded from Firehose. Then, hypermutated samples and the genes with low expression throughout the tumor types were filtered out. Full details of the filtering steps are available in Supp. Figure 1 of the corresponding study^17^. After this preprocessing, the dataset contains somatic aberrations for 11,565 genes in 3110 samples. We perform evaluations on three set of samples: (1) pan-cancer samples, (2) breast cancer samples, (3) lung adenocarcinoma samples. For the PPI network, we use the IntAct network downloaded from https://www.ebi.ac.uk/intact/ on Feb 11, 2019. The interactions with a confidence value less than 0.35 are filtered out. The final network contains 8684 genes and 83,124 edges. To compile reference gene sets for pan-cancer evaluations we use the COSMIC Cancer Gene Census (*CGC*) database^36^. However the *CGC* list lacks a complete annotation of cancer type information. As such, for the breast and lung cancer evaluations, we employ the CancerMine database to construct the corresponding set^37^. CancerMine employs text-mining to catalogue cancer-associated genes through which it also extracts information about cancer types. We compile the list of CancerMine’s breast cancer-associated genes that have at least 3 citations and call it *CMbreast*. Similarly, we call the corresponding list for lung cancer *CMlung* where we use a citation threshold of 2 to retrieve a large enough set of reference genes.

**Figure 1:**
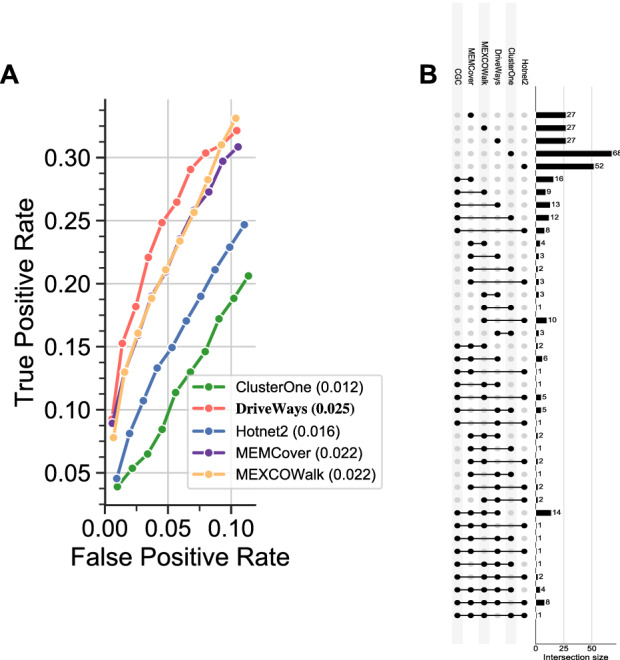
(**A**) ROC curves calculated for *unique_genes* $= 100,200,\ldots,1000$ from the output sets of modules of the methods under consideration. (**B**) Upset graph visualization of overlaps in the sets of top 100 genes output by the methods under consideration.

### Parameter settings

A parameter applied commonly to all the methods under consideration is $\delta _m$ which is set to 3, as this constitutes a nontrivial minimum module size compatible with the problem definition. For DriveWays we find the *t* and *d* setting that maximizes ODMSS. We utilize the BayesOpt procedure implemented in scikit-optimize package to find these values in a time efficient manner^35^. We use the version 0.7.4 with the following setting of the arguments: $n_{calls} = 30$ and $acq\_{func} = EI$. We search for the optimal value of *t* in the range [0.8, 1.2] and the optimal value of *d* in the range [2, 5]. Selected values are available in Table S1 of the Supplementary Information. For ClusterOne, we set the penalty term *p* and the overlap score threshold *w* to their default values, 2 and 0.8, respectively. For Hotnet2, the recommended value of 0.4 is used for the restart probability. Regarding MEXCOWalk, the default values are used for the restart probability ($\beta =0.4$) and the mutual exclusivity threshold ($\theta = 0.7$). For MEMCover, as recommended in the original study, the mutual exclusivity scores are obtained from type-restricted permutation test with all pan-cancer samples. Coverage parameter *k* is set to its default value of 15. $f(\theta )$, which is a parameter that indirectly controls the module sizes in MEMCover, is chosen such that the number of modules with size $<\delta _m$ is minimized.

### Evaluations omitting modularity

Before performing any evaluations with respect to the specific grouping of the output genes into modules, we simply check whether our method recovers more known drivers when each output set under comparison is considered be a single set consisting of the union of the genes in all the output modules provided by each method. We choose to employ the *CGC* genes from COSMIC database as the reference set for defining known drivers. Such a choice is justified by the fact that CGC is a comprehensive and up-to-date source for genes that are causally implicated in cancer. Additionally, our evaluations are consistent with many other cancer driver module identification studies that use CGC as the reference^17,25,26,28^.

One option in evaluating the outputs of different methods by comparing them against the *CGC* reference gene set would be to fix $\delta _s$ across all the methods. However, this would result in varying numbers of unique genes for overlapping and nonoverlapping module finding algorithms making the comparison difficult. Instead, to provide a fair comparison between the methods outputting overlapping modules (DriveWays, ClusterOne, and MEMCover) and those providing nonoverlapping modules (HotNet2 and MEXCOWalk), we obtain the results by varying the total number of unique genes, named *unique_genes*, from 100 to 1000 in steps of size 100. To achieve this for ClusterOne, MEMCover, and DriveWays we take the top ranking modules until the number of unique genes is equal to the *unique_genes*. For Hotnet2 and MEXCOWalk, we choose an edge weight threshold value such that removal of edges below this threshold value results in strongly connected components with total size equal to *unique_genes*. For each method and for each setting of *unique_genes*, we compute the true positive rate (TPR) as the ratio between the number of CGC genes among the top *unique_genes* of the method divided by the total number of CGC genes and the false positive rate (FPR) as the ratio between the number of non-CGC genes among the top *unique_genes* divided by the total number of non-CGC genes. Figure 1A plots the Receiver Operating Characteristic (ROC) curves obtained from each method for the pan-cancer data. We observe that DriveWays performs better than all the other methods. Hotnet2 and ClusterOne perform considerably worse than the other methods. ClusterOne’s poor performance is expected since it does not employ any cancer-related information. The analogous plots for the breast and lung cancer cohorts can be found in Figs. S6 and S10 of the Supplementary Information. For breast cancer, different from the pan-cancer result, MEXCOWalk slightly outperforms DriveWays and MEMCover which are tied as the second best performers. For lung cancer, DriveWays significantly outperforms all the other methods. MEXCOWalk and Hotnet2 together rank the second, and ClusterOne ranks the last. MEMCover outputs only 268 genes when executed on the cohort of lung cancer samples. As such, its AUROC value is not comparable with those of other methods.

We employ an additional comparison using an upset graph representation to assess the degree of overlaps between the output gene sets of different methods; see Fig. 1B. We note that the intersections are obtained without aggregation, that is the row marking two output sets *A*, *B* shows the size of $A\cap B$ excluding $A\cap B\cap C$, for any output set *C*. We observe that ClusterOne and Hotnet2 each outputs a large number of genes that are neither detected by any other method nor found in the CGC reference set. This is expected of ClusterOne since it does not utilize any cancer-related input data and simply serves as a baseline method. The number of candidate driver genes detected only by DriveWays and MEMCover is large, since both have similar optimization goals in terms of coverage and mutual exclusivity of the modules. Similarly, the number of genes detected only by Hotnet2 and MEXCOWalk is also large. For a given method *A*, let the *missing set* of *A* denote the set of CGC genes detected by all methods except *A*. The sizes of the missing sets of DriveWays, MEXCOWalk, MEMCover, HotNet2, and ClusterOne respectively are 1, 0, 0, 4, and 8. The only CGC gene detected by all the methods other than DriveWays is STK11. On the other hand, the number of CGC genes detected only by a single method with respect to the mentioned methods in the same order are respectively, 13, 9, 16, 8, and 12. Analogous upset graph plots for breast and lung cancers are available in the Supplementary Information; see Figs. S6 and S10, respectively.

### Evaluations based on modularity

The goal of the cancer module identification methods is not only recovering the maximum number of known cancer drivers but more importantly providing them as groups of genes that share the same molecular functions or pathways. To retrieve known pathways we utilize three databases: KEGG^38^, Reactome^39^, and BioCarta^40^. To obtain only cancer related pathways, we filter the reference pathways to include only *known cancer genes* and then we remove any resulting pathway with size less than $\delta _m$. Hereafter we denote each such set of reference pathways as $X_Y$, where *X* is the employed pathway database and *Y* is the database of *known cancer genes* employed for filtering *X*. Since providing modules that cover all cancer-related pathways is critical, we set the main goal of the evaluation procedures as recovering each *set of reference pathways*, that is KEGG$_Y$, Reactome$_Y$, or Biocarta$_Y$, where *Y* is *CGC* for the pan-cancer evaluations,*CMbreast* for breast cancer evaluations and *CMlung* for lung cancer evaluations. For the rest of the evaluations, $\delta _s$ parameter is set to $\sum _{\forall M\in D'} |M|$, where $D'$ indicates the corresponding set of reference pathways. This value is 1771 for KEGG$_{CGC}$, 845 genes for KEGG$_{CMbreast}$ and 218 genes for KEGG$_{CMlung}$; 3368, 1416 and 171 genes for Reactome’s respective filtrations; 1173, 626 and 76 genes for Biocarta’s respective filtrations. The desired outputs with the corresponding $\delta _s$ values for different methods can be achieved similar to the approach described in the previous subsection for the *unique_genes*. Note that upon setting $\delta _s$ to match the corresponding value from a specific set of reference pathways for all the methods, each method itself has the flexibility to choose how many unique genes it provides in its output, which in turn is correlated with the sizes of the output modules and the degree of overlaps among them.

#### Statistics on output modules and the sets of reference pathways

**Figure 2:**
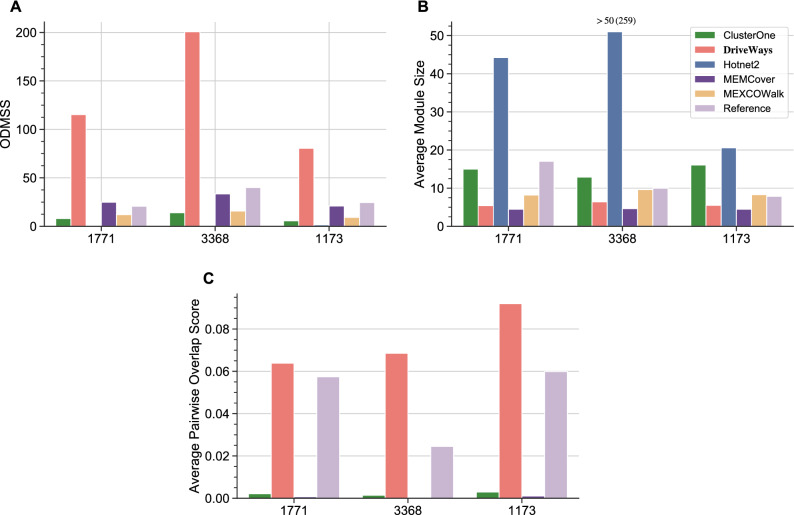
(**A**) ODMSS values for the outputs of all the methods when $\delta _s$ is set to shown values. (**B**) Average module sizes in the outputs of the methods under consideration for the shown $\delta _s$ values. (**C**) Corresponding average pairwise overlap scores.

The first statistic we provide is regarding the main optimization goal of our method, that is the overlapping driver module set score (ODMSS). Figure 2-A shows that DriveWays predicted modules have significantly higher ODMSS values than the output modules of all the other methods. Additionally, the fairly large ODMSS scores observed for sets of reference pathways support the validity of the ODMSS as an objective function. Among the rest of the methods, ClusterOne’s performance is impressive considering that it is not a cancer-specific module identification method; it provides the fourth best performance surpassing Hotnet2. Rather than its module growth procedure, this performance could in part be due to ClusterOne’s seed ranking procedure which is based on the degree of the genes in the network. Using only this seed list as the output set of genes achieves a performance that is even better than that of ClusterOne itself in terms of the CGC overlap evaluations of the previous subsection that considers the union of output modules; see Supplementary Fig. S5. Since most CGC genes also have high coverage scores, it is not surprising to observe that ClusterOne modules result in a high ODMSS value. For this metric, Hotnet2 performs the worst among the considered methods presumably due to its very large modules, as large modules are likely to show poor mutual exclusivitiy.

We next provide certain statistics on the average sizes and the overlap rates of the output modules obtained for the methods under consideration. Figure 2B shows the average module sizes when $\delta _s$ is set to KEGG$_{CGC}$, Reactome$_{CGC}$, and Biocarta$_{CGC}$ sizes. The same plot also includes the average module sizes of the three sets of reference pathways themselves for comparison. We observe that Hotnet2 has the largest average module size which is significantly larger than those of the other methods. The second and the third largest average module sizes are obtained with ClusterOne and MEXCOWalk outputs, respectively. MEMCover and DriveWays have similar average module sizes that are smaller than the others. Further detailed statistics on the number of output modules and the number of unique genes in all the output modules pertaining both to the outputs of alternative methods and also to the actual sets of reference pathways themselves can be found in Tables S2 and S3 of the Supplementary Information.

We quantify the degree of overlap among the output modules by calculating a *pairwise overlap score*, as previously defined in^41^. For two modules $M_i$ and $M_j$, the pairwise overlap score is calculated as, $\frac{|M_i \cap M_j|^2}{|M_i| \times |M_j|}$. We calculate the sum of the pairwise overlap scores for all pair of modules and normalize it by dividing by the number of all such pairs. Figure 2C shows the resulting average pairwise overlap scores of all the methods and the sets of reference pathways. Here, Hotnet2 and MEXCOWalk are excluded as they provide non-overlapping modules. We observe that modules of DriveWays overlap with each other more compared to the modules of other methods and that this overlap is quite similar to the overlap of the sets of reference pathways themselves.

ODMSS values and analogous statistics on the average sizes and the overlap rates of the output modules are also calculated for breast and lung cancer; see Figs. S7 and S11 of the Supplementary Information for the relevant plots.

#### Definitions of Quality measures for evaluations based on modularity

A given set of predicted modules is evaluated by assessing how well they match and cover a set of reference pathways. Let $\{M_1, \ldots , M_m\}$, $\{R_1, \ldots , R_n\}$ denote the set of predicted modules and the set of reference pathways, respectively. We introduce three measures to quantify the similarity between a predicted module $M_i$ and a reference pathway $R_j$.

##### Overlap score

We calculate the overlap score between a module and a pathway as the pairwise overlap score defined in the previous subsection, replacing $M_j$ with the reference pathway $R_j$ in the formula.

##### Hypergeometric test q-value

 A hyper-geometric enrichment test is used to evaluate the significance of the intersection of $M_i$ with $R_j$. Adjusted p-values (also called q-values) are calculated with False Discovery Rate (FDR) correction^42^.

##### GO consistency score

 This score has been previously employed for the evaluation of PPI network alignment algorithms^43^. We employ the go-basic.obo file from http://geneontology.org on June 26, 2019. We restrict the gene annotations to level 5 of the GO hierarchy by ignoring the higher-level annotations and replacing the deeper-level category annotations with their ancestors at the restricted level. We call the resulting terms as the *standardized GO terms*. Let $GO_{M_i}$ and $GO_{R_j}$ denote the union of the standardized GO terms obtained from the GO annotations of the genes in $M_i$ and $R_j$, respectively. GO consistency score of $M_i$ and $R_j$ is defined as, $GO(M_i,R_j) = \frac{|GO_{M_i} \cap GO_{R_j}|}{|GO_{M_i} \cup GO_{R_j}|}$.

Rather than identifying the enriched pathways for each module separately, we use an evaluation procedure which ensures that the set of predicted modules as a whole provides a good match to the whole set of reference pathways. To this end, the first metric we use is based on *Maximum Weighted Maximum Cardinality Matching (MWMCM)*. To identify MWMCM, we first create a bipartite graph containing nodes corresponding to the predicted modules on the one side and nodes corresponding to the reference pathways on the other side. The edge weights between a predicted module $M_i$ and a reference module $R_j$ are computed using one of the three similarity measures defined above. The overlap score and the GO consistency score of $M_i$ and $R_j$ can each be directly used as edge weights between the corresponding nodes of the bipartite graph. To use the q-values as edge weights, we transform them by taking the $-log_{10}$ of the values so that larger ones correspond to better matches. Also, if the q-value is $>0.05$, we instead assign zero as the edge weight as this corresponds to a non-significant match. Once the bipartite graph is formed, we find the MWMCM; that is, we find a subset of edges such that each predicted module and reference pathway is incident on at most one selected edge, the number of such selected edges is maximum (maximum cardinality matching), and the sum of the weights of selected edges is maximized among all maximum cardinality matchings. Lastly, we calculate the average weight of the edges in the resulting matching. We call this score Maximum Matching Ratio (MMR), as in^31^; see Supplementary Fig. S10 for a plot depicting how MMR is computed. We emphasize the fact that we employ a complete bipartite graph where zero-weight edges are also included, since excluding such edges could provide misleading results. For instance, consider a scenario where only one of the output modules is a perfect match to a reference pathway and the remaining modules show no similarity under any defined measure with any member of the set of reference pathways. When there is no similarity, the weights of edges connected to those modules would be zero. If zero-weight edges are removed the hypothetical method providing such an output would get a perfect MMR value of 1 even though only one of its predicted modules can be considered “good”; see Supplementary Fig. S11 for a toy example. On the other hand, calculation of MMR with zero-weight edges ensures that every predicted driver module is enriched for a functional pathway important for cancer.

#### Recovering sets of cancer-associated reference pathways

**Figure 3:**
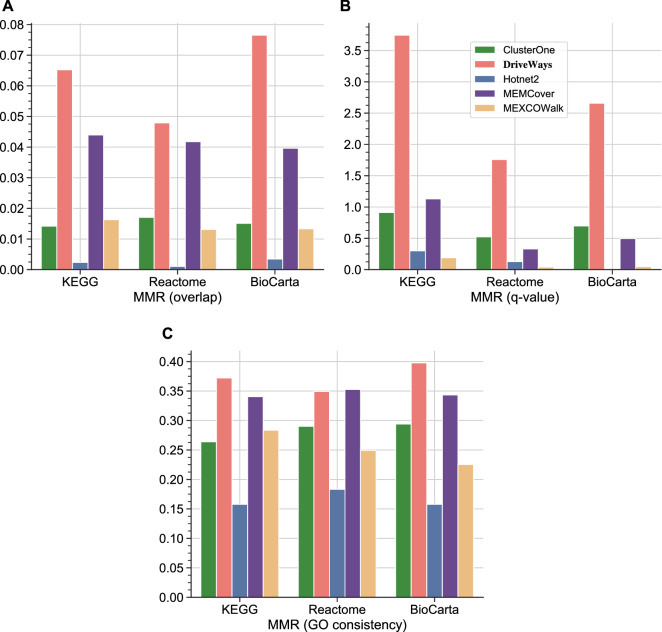
MMR scores of all methods calculated with three similarity metrics: (**A**) Overlap score (**B**) Hypergeometric test q-values (**C**) GO consistency. The set of reference pathways at the x-coordinate of each plot correspond to KEGG$_{CGC}$, Reactome$_{CGC}$, and Biocarta$_{CGC}$, from left to right.

Figure 3 displays the MMR results calculated with the three similarity measures. MEMCover has a slightly better MMR score than DriveWays under the GO consistency similarity measure when Reactome$_{CGC}$ is used as the reference. In all the other evaluations, DriveWays gives higher MMR values than the competing methods. MEMCover ranks the second in most cases except for the MMR score under the q-value similarity measure when Reactome$_{CGC}$ or Biocarta$_{CGC}$ is used as the reference. In these two cases, ClusterOne performs better than MEMCover. Hotnet2 performs the worst in all evaluations. In particular, Hotnet2’s MMR scores under the q-value measure are close to zero presumably due to its large-sized modules.

Next, we utilize the precision and recall metrics to evaluate the predicted modules. A modified version of these metrics have been previously used in evaluating the quality of the predicted modules^30,44^. To evaluate the precision of a method, for each of its predicted module $M_i$, we find the *best match* in the set of reference pathways using one of the three similarity measures, overlap score, q-value, or GO consistency score. We evaluate recall similarly, but this time we find the *best match* of each reference pathway $R_j$ among the predicted modules using one of the similarity measures. Identification of the best match for each predicted module and for each reference pathway is illustrated with a toy example in Section 1.4 of the Supplementary Information. We plot the distribution of the best match scores across all the predicted modules and across all the reference pathways. Supplementary Fig. S2 shows these distributions for each similarity metric, for each method, and for each set of reference pathways. We observe that the best match scores of DriveWays-predicted modules are significantly higher than the best match scores obtained with the other methods for all similarity metrics and for all sets of reference pathways. In terms of the best match scores of the set of reference pathways, DriveWays performs better in the majority of the cases with few exceptions. MEMCover performs slightly better than DriveWays under the overlap score similarity measure with respect to the Reactome$_{CGC}$ set of reference pathways. Similarly, MEMCover performs better under the GO consistency similarity measure with respect to the Reactome$_{CGC}$ and BioCarta$_{CGC}$ sets of reference pathways. DriveWays’s slightly worse performance in terms of recall in these cases can be attributed to the relatively high pairwise overlaps of its output modules. Since $\delta _s$ is fixed, DriveWays outputs smaller number of unique genes as compared to the other methods and also as compared to the sets of reference pahtways. This is why some sets of reference pathways could have low best match scores since the genes in those reference pathways do not exist in the output modules of DriveWays. However, the performance difference between DriveWays and the rest of the methods in terms of precision dominates the performance difference between MEMCover and DriveWays in terms of recall. To illustrate this, we also compute an aggregate score by finding the average best match score across the predicted modules and the average best match score across the reference pathways. F1 scores obtained by the product of these two average values are shown in Supplementary Table S4. DriveWays has the highest F1 score in all the experiments illustrating its overall superiority with respect to precision and recall.

We repeat the same evaluations for the breast cancer and lung cancer. Almost all the results regarding the pan-cancer data discussed here apply similarly in this setting as well; see Sections 1.2 and 1.3 as well as Tables S5–S6 of the Supplementary Information for detailed results.

#### Top DriveWays modules compared to their matches

We next investigate our top modules and their matched reference pathways more closely under the pan-cancer setting. We first look at our top ten modules and the KEGG$_{CGC}$ reference pathways matched to them in the context of MWMMC. First of all, we observe that all top 10 modules are incident on the set of edges selected for MWMMC. We further explore the reference pathways that are connected to the top 10 modules through these selected edges. Five of the ten such reference pathways directly correspond to a pathway of a specific cancer type: *Non small cell lung cancer*, *Bladder cancer*, *Glioma*, *Pancreatic cancer* and *Endometrial cancer*. Among the other matched reference pathways, *Cell cycle* pathway and the *p53 signalling pathway* are also strongly associated with cancer. We observe that the matches to *Cell cycle* and the *Non small cell lung cancer* pathways have the highest edge weights. For both matches, our predicted modules consist of five genes all of which also appear in the matched reference pathways. Another interesting match is observed between our seventh ranking module and the *Bladder cancer* pathway. Our predicted module contains six genes, four of which appear in the *Bladder cancer* pathway. VHL, a well known tumor-suppressor, is among the two genes that appear in our predicted module but not in the *Bladder cancer* pathway. Interestingly, among the cancer types in pan-cancer cohort, bladder cancer ranks second after renal cell carcinoma in terms of VHL’s mutation frequency.

We also explore the matches that are found within the contexts of precision and recall under the same setting. For the former, we explore the best matching reference pathways of top 20 predicted modules of DriveWays. Among the best matched KEGG$_{CGC}$ pathways, we observe *Pathways in cancer* nine times, *Cell cycle* four times, *ERBB signaling pathway* three times, *p53 signalling pathway* twice, *MAPK signaling pathway* and *TGFB signaling pathway* once. In terms of recall, we identify the cancer related pathways in KEGG$_{CGC}$ and find their best matches among the predicted modules of DriveWays and MEMCover; the two methods that can identify overlapping cancer driver pathways. When compared with MEMCover, except for the *Colorectal cancer pathway*, DriveWays’s predicted modules result in a better overlap score with cancer related KEGG$_{CGC}$ pathways. Overall these results show that top DriveWays modules are enriched for cancer associated pathways in KEGG.

Lastly, we investigate the known cancer genes that occur in multiple pathways and check whether any such gene is only recovered by DriveWays. We find that Neuregulin (NRG1) is a known cancer gene and is only identified by DriveWays. Neuregulin (NRG1) is expressed in numerous isoforms and has important roles in multiple signalling mechanisms^45^ as well as in cancer progression^46^. Accordingly, NRG1 appears eight times in Reactome$_{CGC}$ and three times in our predicted output modules. This result shows the benefit of considering multiple functions of a single gene.

#### Novel candidate driver genes and modules of DriveWays

We inspect the top twenty DriveWays modules and the genes therein under the pan-cancer setting in more detail. We focus on the novel candidate driver genes, that is those that are not labelled as cancer genes by the CGC reference dataset but that reside in the KEGG pathways that correspond to the best matches of their respective modules. Throughout the analysis we focus on the genes with more than 10 mutations in the relevant cohort. There are 4 such genes. These are LTBP1, SMC3, SMC1A, and FLNA. The best match of the module containing LTBP1 is the *TGFB signaling pathway*. It is mutated in 59 samples and interacts with KAT6A and KAT6B which also reside in the same output module as LTBP1 and which are part of the CGC reference gene set. Indeed LTBP1 has been reported to play an important role in cancer in several previous studies, including its role as a potential biomarker in ovarian cancer^47^ and its role in enhancing metastatic behavior in breast cancer^48^. The gene SMC3 which is mutated in 35 samples appears in the same DriveWays module as MYC, MDM2, TP53, and APC, all of which are known cancer genes part of the reference CGC. Among the KEGG pathways under consideration, the module containing SMC3 has the best match with the *Cell cycle* pathway. On the other hand, SMC1A, similar to SMC3, is another cohesin subunit missing from the CGC. It is mutated in 44 samples and it appears in a DriveWays module together with CDKN2A, TP53, MYC, ATM, and VHL, all of which are CGC genes. Evidence from previous studies suggests that the chromatid cohesion defects may underlie chromosome instability and tumour development, emphasizing the role of SMC3 and SMC1A as candidate drivers^49,50^. Finally, FLNA, an actin cross-linking protein is mutated in 51 samples and shares a DriveWays module with IKBKB, TP53, and APC. Each of the latter genes is a CGC gene, whereas FLNA is not. The module itself has the best match with the *MAPK signaling pathway*. Further support of the DriveWays’ choice of FLNA as a candidate driver is found in various recent studies demonstrating its role in tumor progression in a wide range of cancer types^51–53^. Repeating the same analysis under the breast cancer setting we find that PIK3R1, CDK4, CDK6, and SF3B1 satisfy the criteria for driver candidacy, that is each one appears among the top twenty DriveWays modules, does not exist in the reference gene set *CMbreast*, but does exist in the KEGG pathway corresponding to the best match of its module. PIK3R1 is in a DriveWays module containing PIK3CA and ERBB2, both of which are associated with breast cancer through the *CMbreast* reference set. It is noteworthy that the mutation frequency of PIK3R1 in the cohort is quite low; only 19 samples contain mutations in the gene. This demonstrates the effectiveness of the proposed model of DriveWays in detecting rare drivers through the insightful incorporation of the mutual exclusivity concept. Indeed a recent survey of the landscape of somatic mutations in Chinese breast cancer patients suggests evidence supporting the driver candidacy of PIK3R1 in oncogenesis^54^. Furthermore the same study reports a pattern of mutual exclusivity for driver mutations in PIK3CA and PIK3R1 in the relevant cohort, which is attributed to the hyperactivity of the PI3K pathway. On the other hand, CDK4/6 which are amongst the genes rarely mutated in our cohort of study, have been known to play a key role in the proliferation of both normal breast epithelium and breast cancer cells, and therapies based on CDK4/6 inhibitors are suggested for certain types of breast cancer^55^. The mutations of the other candidate suggested by DriveWays, SF3B1, which is another less frequently mutated gene in our cohort with only 14 mutations, has been recently found to promote tumorigenesis through MYC stabilization^56^. Interestingly, as yet another evidence of DriveWays’ success in not only choosing potential driver gene candidates but also in placing them into relevant modules, DriveWays places SF3B1 in the same candidate driver module as MYC. Finally, the analogous analysis under the lung cancer setting provides us with a single candidate provided by DriveWays, but nonexistent in the relevant reference set of *CMlung*. That candidate is MDM2. DriveWays exclusively places MDM2 together with TP53 in several of its top modules. This is no surprise due to the quite well-known role of MDM2 as an important regulator of the p53 pathway and its effects on the anti-tumorigenic activity of the p53^57^.

## Discussion

The prevalent role of a single protein in multiple functional pathways is usually an overlooked fact among cancer driver module identification methods, most of which provide set of nonoverlapping modules of genes driving cancer. We provide the definition of an optimization problem that models possibly overlapping modules of driver genes and a method, DriveWays, to efficiently identify driver modules in cancer according to this novel problem definition. DriveWays incorporates network connectivity, mutual exclusivity, and coverage information to identify overlapping cancer driver modules. It does not require any additional parameters, other than the desired minimum size of a module and the sum of the sizes of all the modules, both of which should be intuitive properties for cancer biologists. In addition to methodological contributions, our work also proposes novel evaluation metrics suitable for fair comparison of methods that provide possibly overlapping cancer driver modules. This contribution is valuable as the majority of existing evaluation strategies for cancer module finding methods ignore the specific grouping of genes to modules by collapsing all the modules into a single set.

Comparing against four state-of-the-art methods, we demonstrate the ability of DriveWays to identify modules enriched with known cancer genes, and also enriched for curated pathways containing only known cancer driver genes. As far as the the fairness of the provided comparison studies is concerned, we refer to three general criteria as discussed in Boulesteix *et al.*^58^: Choice of methods and method parameters, choice of evaluation criteria, and choice of data sets. Regarding the first criterion, we note that the method most suitable for comparison against DriveWays is MEMCover, as its assumed input and output match closest to that of the definition of the ODMIC problem; the input consists of mutations data and the PPI network, and the output is a set of possibly partially overlapping sets of genes. Hotnet2 and MEXCOWalk on the other hand assume the same type of input but prohibit any overlaps in the output set of modules. The former is a quite popular benchmark method in cancer driver module discovery studies and the latter is a fairly recent representative shown to outperform competitors with respect to several performance criteria. Although the output data of ClusterOne is of the same type as that of DriveWays and MEMCover, in terms of the assumed input it only makes use of the PPI network. This makes it a good candidate to serve as a baseline method to compare against the rest of the four methods under comparison. In terms of the various parameters passed to the methods under comparison, we employed the default settings as suggested by each approach. With regard to the choice of evaluation criteria, we made a careful distinction between those that omit the modular separation of the output set of genes and those that take this fact into account. The former focuses on the area under the curve as the main evaluation metric, in line with the citerion suggested for supervised classification algorithms^58^. Naturally, the baseline algorithm, ClusterOne, performs quite poorly as compared to the rest of the methods which make use of contextual knowledge in the form of mutations data. On the other hand, the second type of evaluation criteria are based on modularity and place an emphasis on the way the output set of genes are separated into distinct modules, as well as the set of genes themselves. Furthermore, these criteria test the overlapping nature of the produced output modules by measuring how well the produced modules mimic the functional pathways induced only by known cancer driver genes, as such pathways are known to exhibit overlaps upto a certain degree. Thus the performance of the baseline method ClusterOne is similar to or in some instances even better than those of Hotnet2 and MEXCOWalk, as the latter approaches explicitly suppress any overlaps in their output modules. This reflects the fact that such criteria are not affected by any bias possibly introduced by the commonly employed area under the curve metrics. Finally, with regard to the choice of employed datasets, we note that both the TCGA data and the IntAct PPI network are two of the most common datasets among their respective data types. Additionally, any employed data preprocessing is applied pervasively so that all the methods under consideration are passed exactly the same preprocessed data as input.

Finally, we demonstrate the robustness of DriveWays results by varying several relevant parameters. With respect to the $\delta _m$ parameter, we try the settings 4 and 5 in addition to the default value of 3. As a second robustness test we vary the set of samples by creating 100 bootstrapped resamples and run DriveWays on these samples. Finally, as the last robustness test, we investigate the effects of the employed PPI by using a different PPI network, the HINT+HI2012 network^59,60^. For all the robustness experiments, we observe negligible performance differences when compared to the original results of DriveWays with respect to almost all the evaluation settings under consideration; see Supplementary Information, Section 1.5 for detailed figures depicting the evaluation results.

As part of possible future research directions, we note that DriveWays can be improved in several ways. Current implementation utilizes only mutation data to enable comparison with many existing approaches. One direction is to incorporate additional types of genomic data from TCGA project such as gene expression, DNA methylation etc. One limitation of using bulk expression or sequencing data is the presence of non-cancerous cells in bulk samples. Recently published single-cell RNA-seq datasets can be utilized to account for intra-tumor heterogeneity. Currently, these are available for a small number of patients. However, one can use deconvolution approaches to analyze bulk RNA-seq data in light of single-cell RNA-seq measurements to infer sample level cell-type specific gene expression profiles^61^. A related promising direction is to utilize single-cell DNA-seq datasets for detection of mutations as they become available for large number of samples. Lastly, DriveWays’s performance is directly affected by the accuracy of PPI data. As such, another direction for improvement is to use tissue-specific PPI based on the cancer type.

Apart from identification of novel cancer drivers, DriveWays can also be useful in elucidating the functions and roles of these drivers as it outputs not solely a list of candidate genes but rather a compartmentalized set consisting of modules of genes acting together in their roles as cancer drivers. Finding the reference pathways that best match to these modules would provide hints on the mechanisms of actions of the involved candidate driver genes. This could also be useful in developing cancer therapies. For instance, a predicted cancer gene that occurs in several cancer driver pathways could be a good target for drug treatment. Lastly, the Driveways algorithm may be of use not only in disease studies, but also in various application areas involving overlapping community detection. DriveWays would require very little application-dependent modification for such uses. Those would be limited only to providing proper definitions of module score (MS) and Inequality (1).

## Supplementary information

Supplementary Information 1

## Data Availability

The data, the source code, and useful scripts are available at: https://github.com/abucompbio/DriveWays. Supplementary data are available at *Scientific Reports*.
