# Supplementary material for: DriveWays: a method for identifying possibly overlapping driver pathways in cancer
Source: Sci Rep. 2020 Dec 15;10:21971. doi: 10.1038/s41598-020-78852-8 (PMC7738685; doi:10.1038/s41598-020-78852-8)
Supplement: Supplementary file 1 — Supplementary Information 1 [file 41598_2020_78852_MOESM1_ESM.pdf]

Supplementary Material for ”DriveWays : A Method for Identifying Possibly Overlapping Driver Pathways in Cancer”

Ilyes Baali[1], Cesim Erten[2,*], and Hilal Kazan[2,*]

[1] Electrical and Computer Engineering Graduate Program, Antalya Bilim University, Antalya, 07190, Turkey
[2] Department of Computer Engineering, Antalya Bilim University, Antalya, 07190, Turkey
[*] corresponding authors: cesim.erten@antalya.edu.tr,hilal.kazan@antalya.edu.tr

1.1 The Module Score Definition

The functions COV and MEX are in many cases conflicting, in the sense that large coverage can be obtained at the expense of mutual exclusivity and vice versa. Therefore, we combine the two functions in a product form and define the *module score* of M as, $MS(M) = COV(M) \times MEX(M)$. An instance depicting the advantage of such a product form formula over an additive function can be found in Figure **??**. In this instance there are a total of 100 samples. The solid vertical black line represents the set of all samples. Each red/blue line represents a gene. The vertical span of each of the red/blue lines reflected on the black line indicates the set of samples where a gene represented by the red/blue line is mutated. Each red line is of length 45, that is there are 45 patients for which a gene represented by a red line is mutated. Each red line is 5 units below the previous red line, indicating that only the first 5 patients for which the gene represented by the previous red line do not have mutations in the gene represented with the current red line. Each blue line is of length 28 and each blue line is 13 units below the preceding blue line. The set of genes represented by the red lines is module A and the set of genes represented by the blue lines is module B. 54% of all the samples are *covered* by the module B. In other words, module B 'explains' more than half of the data with only a quarter of the size of (number of genes) module A. Thus intuitively module B is a *better* module than the module A. However comparing the scorings, we can observe that the module A has an additive score of 1.185, a larger score than 1.183, the score obtained by module B. On the other hand, module A has a score of 0.185 in terms of the product-form formula employed by our definition of MS, a much smaller score than that of module B, which stands at 0.347.

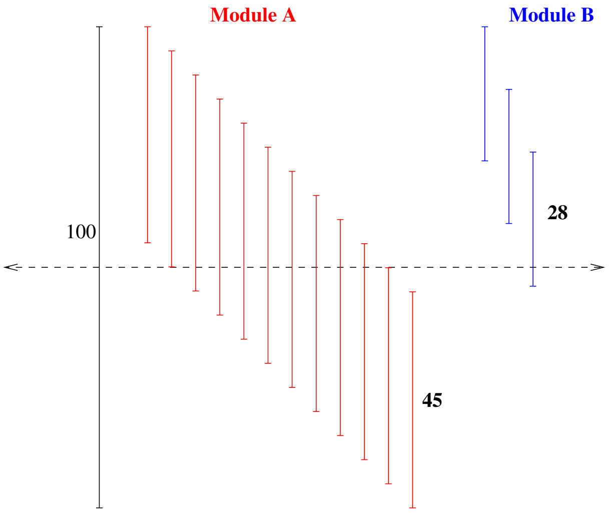

Figure S1: An instance of the ODMIC problem where the product-form formula for the module score is intuitively better than the additive formula.

Theorem 1.1. *Overlapping driver module identification in cancer is NP-hard.*

Proof. The transformation is from *Set Packing* which is NP-complete [?]. In the Set Packing problem, given a collection C of finite sets and a positive integer $K \leq |C|$, the problem is to find out whether C contains at least K mutually disjoint sets. The problem is NP-hard even when the size of each set is at most 3, which can easily be extended to the setting where the size of each set is exactly 3. Given an input to the Set Packing problem within this setting in the form of K and C such that for each $S \in C$, $|S| = 3$, we generate G as a complete graph on $|C|$ vertices, corresponding to the such that each finite set in C corresponds to a set of samples S_i for which gene g_i is mutated. We set both δ_s and *min_module_size* to K. The answer to the Set Packing problem is Yes, if and only if the maximized score of the overlapping driver module identification in cancer problem is exactly $\frac{3\times K}{|\bigcup_{\forall g_i \in V} S_i|}$.

Note that the same reduction is employed in the NP-hardness proof of the problem considered in [?], within the nonoverlapping setting. The reduction works for our setting as well, since the reduction makes sure the output of the overlapping driver module identification in cancer problem produces a single module of size K. □

Lemma 1.2. *There is an optimum solution D of the ODMIC problem on input instance $\prec G, S, \delta_m, \delta_s \succ$, where $|M| < 2\delta_m$, $\forall M \in D$.*

Proof. Let D be an optimum solution. We show that any $M \in D$ with $|M| \geq 2\delta_m$ can be split into two smaller modules M_1, M_2, each satisfying the δ_m constraint, such that $MS(M_1) + MS(M_2) \geq MS(M)$. Let u_1, s_1 denote respectively $|\bigcup_{\forall g_i \in M_1} S_i|$ and $\sum_{\forall g_i \in M_1} |S_i|$. Let u_2, s_2 denote analogous values for M_2. Since $u_1 + u_2 \geq |\bigcup_{\forall g_i \in M} S_i|$, we need to show that $\frac{u_1^2}{s_1} + \frac{u_2^2}{s_2} \geq \frac{(u_1+u_2)^2}{s_1+s_2}$, which holds trivially since $(u_1 s_2 - u_2 s_1)^2 \geq 0$. □

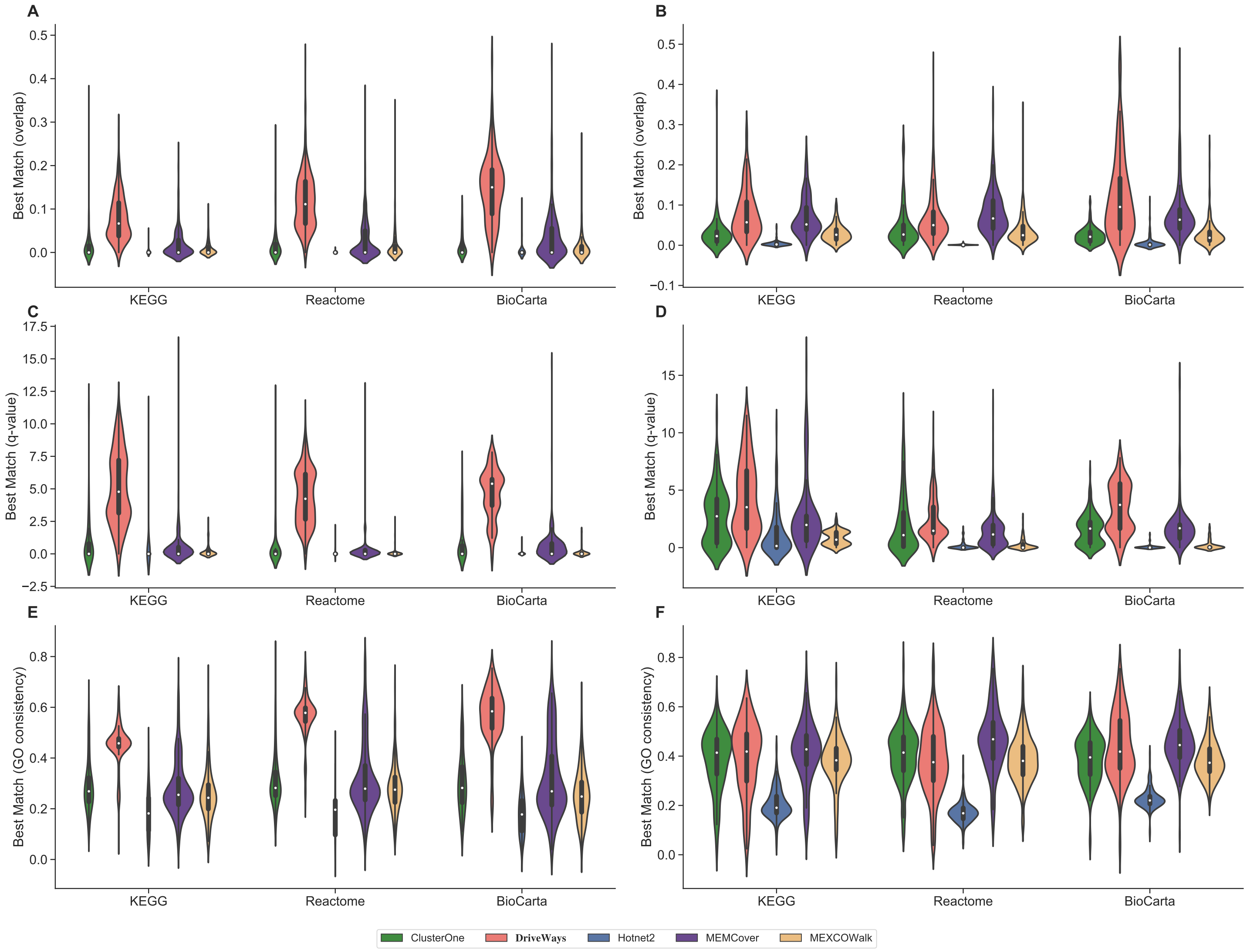

Figure S2: Evaluation of precision by comparing distribution of best match scores for each predicted module when pan-cancer samples are used as input; scores are calculated with (A) overlap score (C) q-value and (E) GO consistency. Evaluation of recall by comparing distribution of best match scores for each reference pathway when pan-cancer samples are used as input; scores are calculated with (B) overlap score (D) q-value (F) GO consistency.

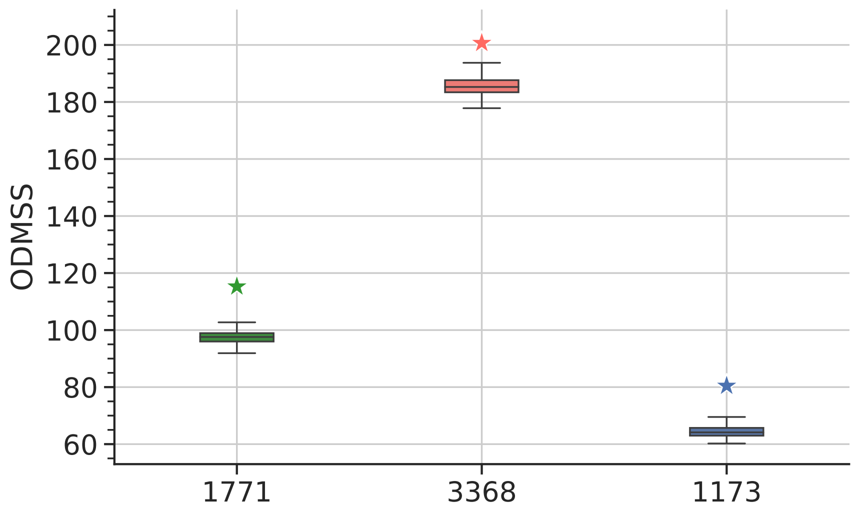

Figure S3: ODMSS results when DriveWays is run with a random seed list. ⋆ represents the results when the original seed list is used in DriveWays runs.

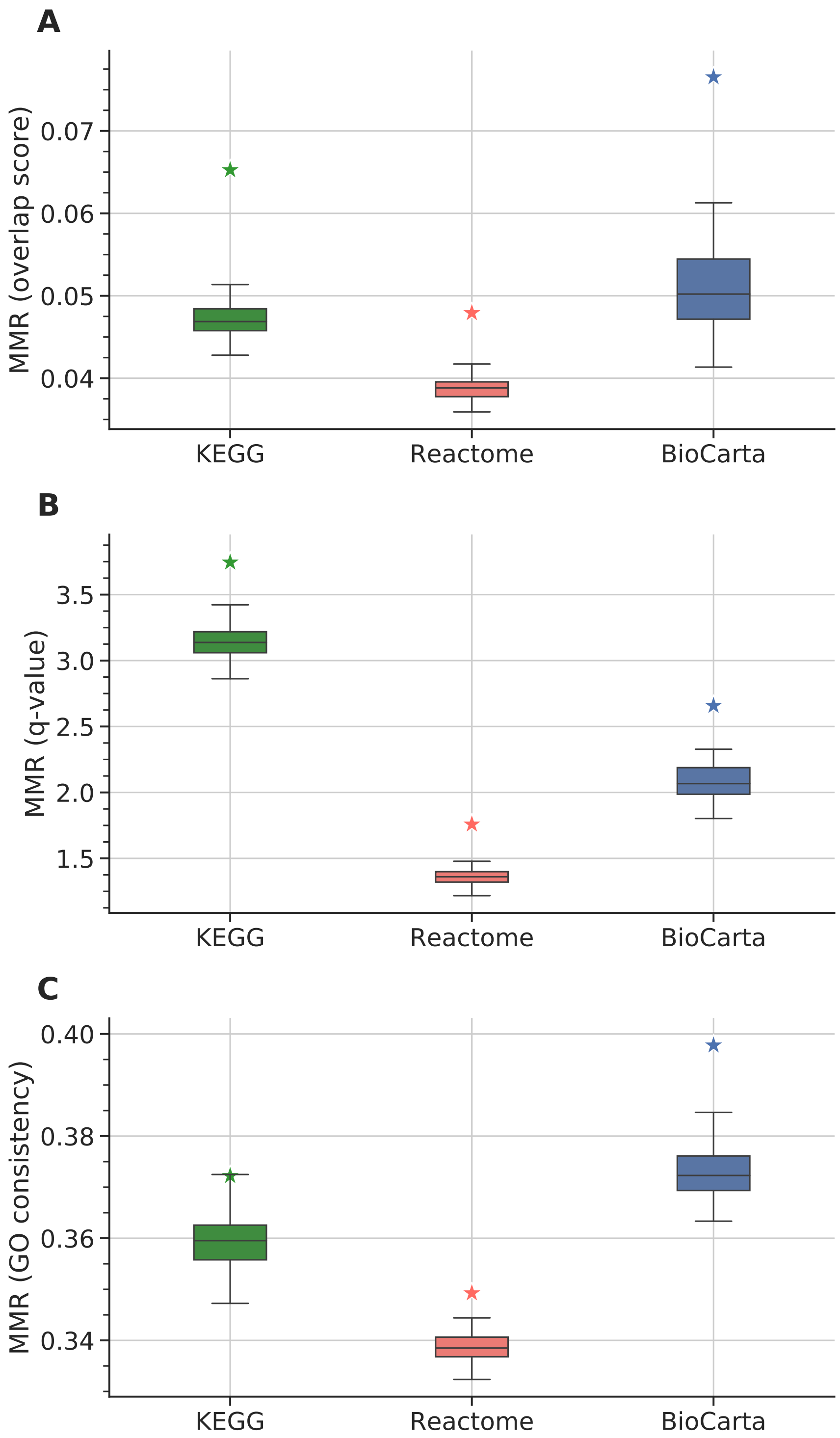

Figure S4: MMR scores when DriveWays is run with a random seed list. A) Overlap score B) Hypergeometric test q-values C) GO consistency. ⋆ represents the scores when the original seed list is used in DriveWays runs.

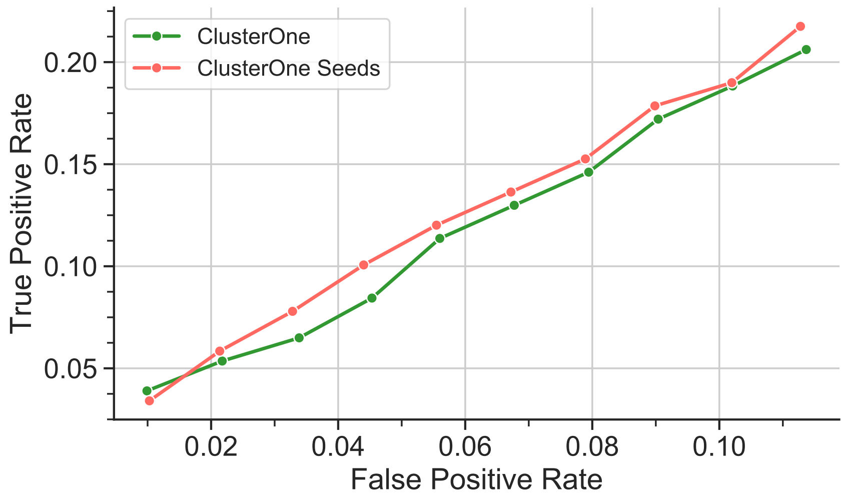

Figure S5: CGC overlap evaluation of ClusterOne output modules and ClusterOne seeds when pan-cancer samples are used as input. ROC curves are calculated for $unique\_genes = 100, 200, ..., 1000$. Note that ClusterOne seeds refer to using seeds directly without any module growth process.

1.2 Results on the BRCA dataset

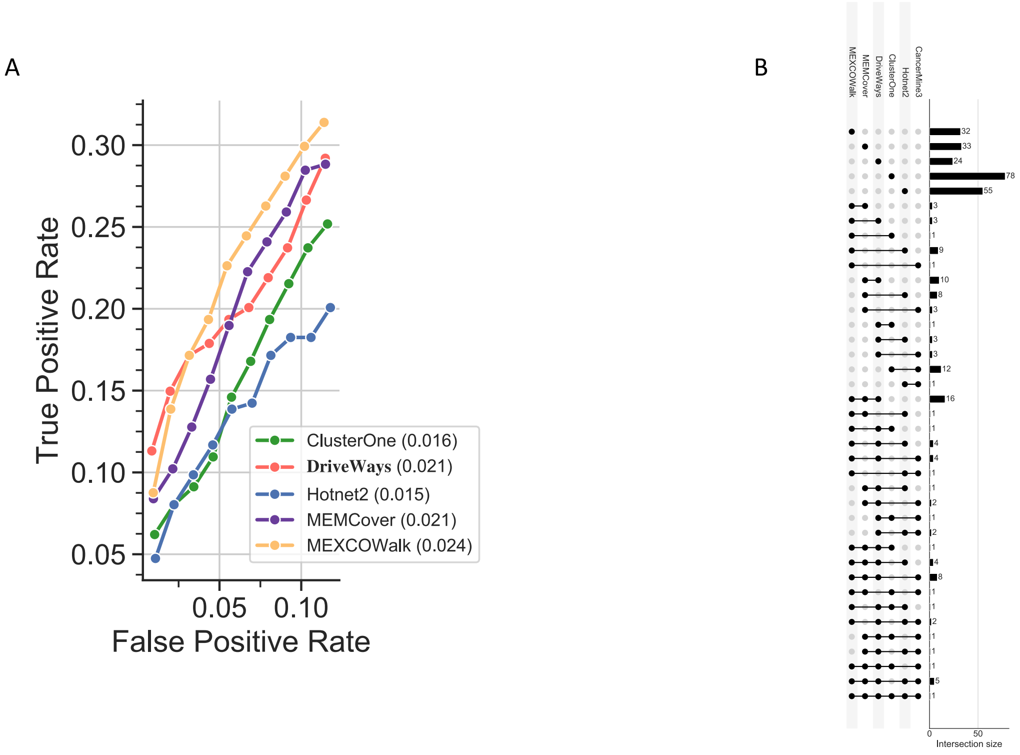

Figure S6: A) ROC curves calculated for *unique_genes* $= 100, 200, ..., 1000$ from the outputs of considered methods when breast cancer samples are used as input and *CMbreast* is used for reference. B) Upset graph visualization of overlaps in the sets of top 100 genes output by the methods under consideration.

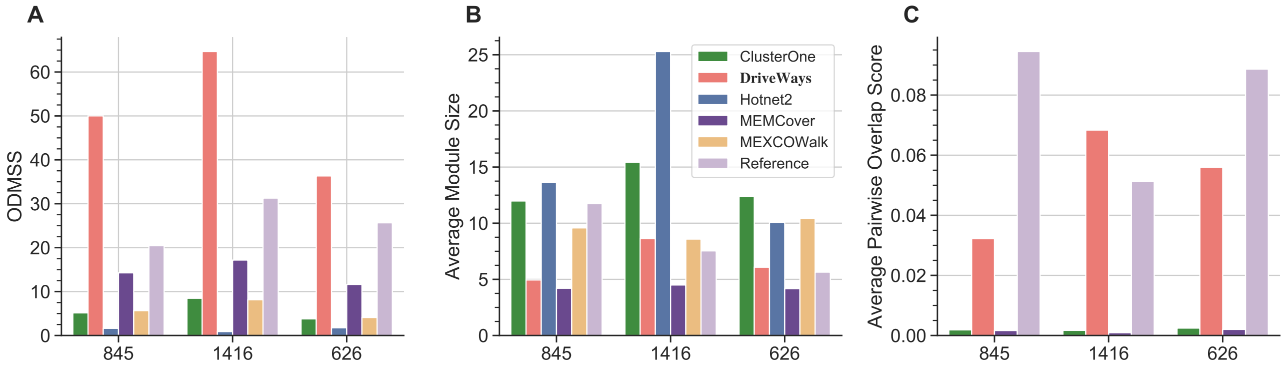

Figure S7: A)Overlapping driver module set score (ODMSS) obtained from breast cancer samples when δ_s parameter is determined based on the total size of KEGG$_{CMbreast}$, Reactome$_{CMbreast}$ and BioCarta$_{CMbreast}$ pathways, respectively. B) Average module sizes in the outputs of the methods under consideration for the shown δ_s values obtained from breast cancer samples. C) Corresponding average pairwise overlap scores.

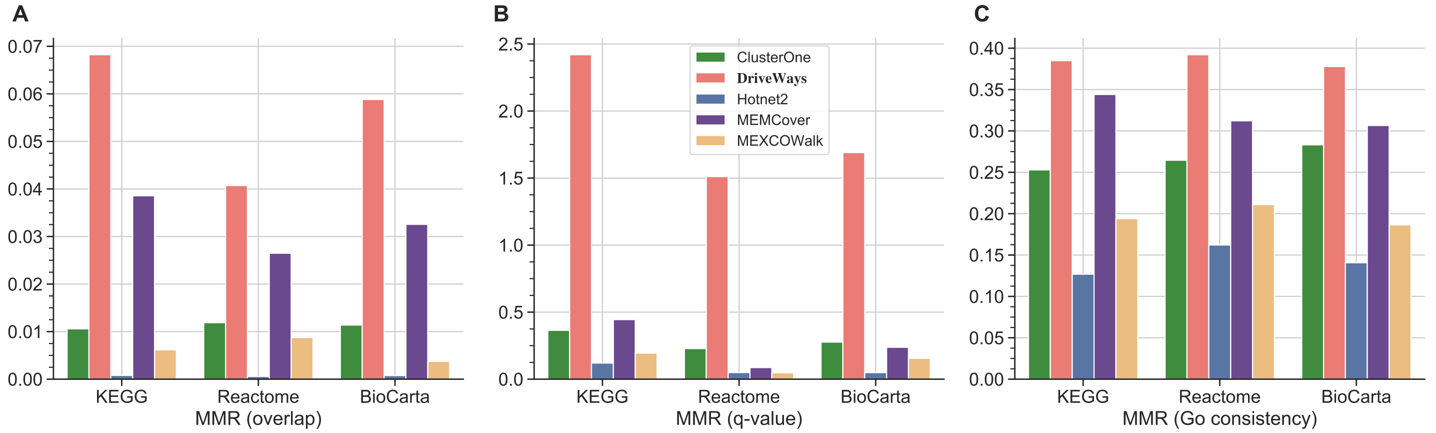

Figure S8: MMR scores of all methods calculated with three similarity metrics: A) Overlap score B) Hypergeometric test q-values C) GO consistency. The set of reference pathways at the x-coordinate of each plot correspond to KEGG$_{CMbreast}$, Reactome$_{CMbreast}$, and Biocarta$_{CMbreast}$, from left to right.

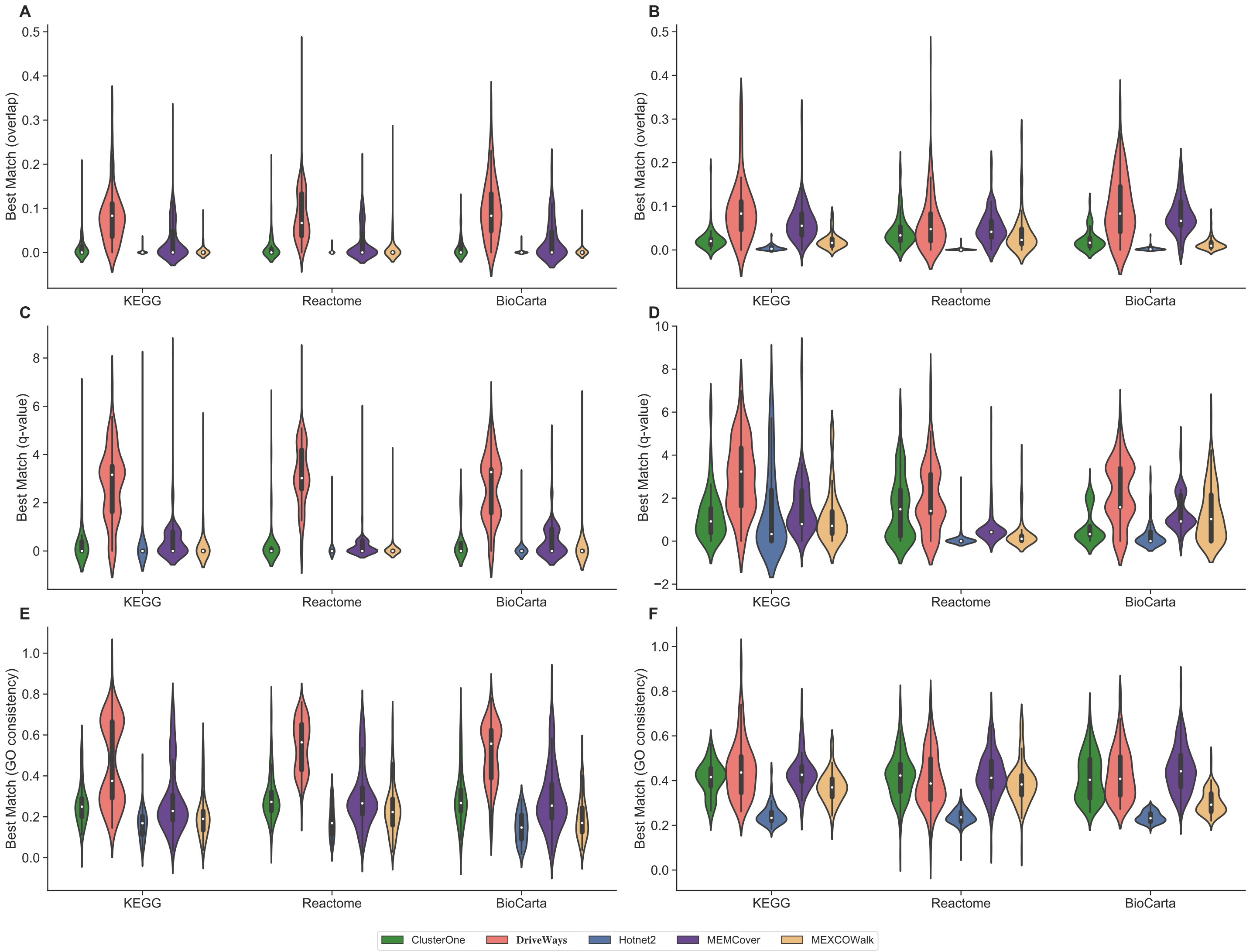

Figure S9: Evaluation of precision by comparing distribution of best match scores for each predicted module when breast cancer samples are used as input; scores are calculated with (A) overlap score (C) q-value and (E) GO consistency. Evaluation of recall by comparing distribution of best match scores for each reference pathway when breast cancer samples are used as input; scores are calculated with (B) overlap score (D) q-value (F) GO consistency.

1.3 Results on the LUAD dataset

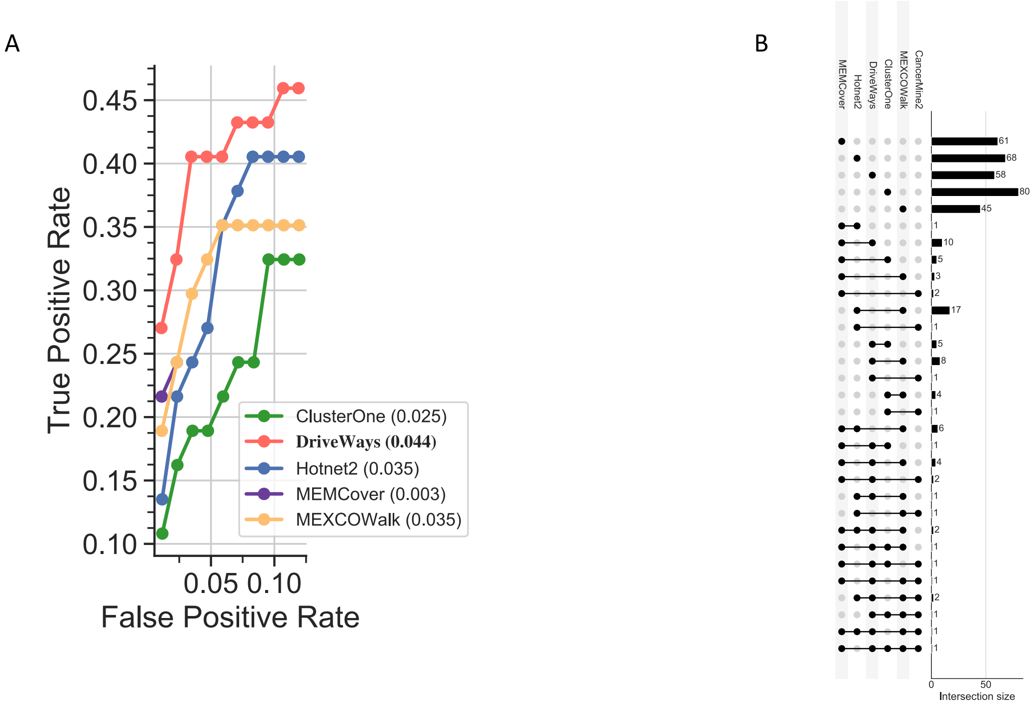

Figure S10: A) ROC curves calculated for $unique\_genes = 100, 200, ..., 1000$ from the outputs of considered methods when lung cancer samples are used as input and *CMlung* is used for reference. B) Upset graph visualization of overlaps in the sets of top 100 genes output by the methods under consideration.

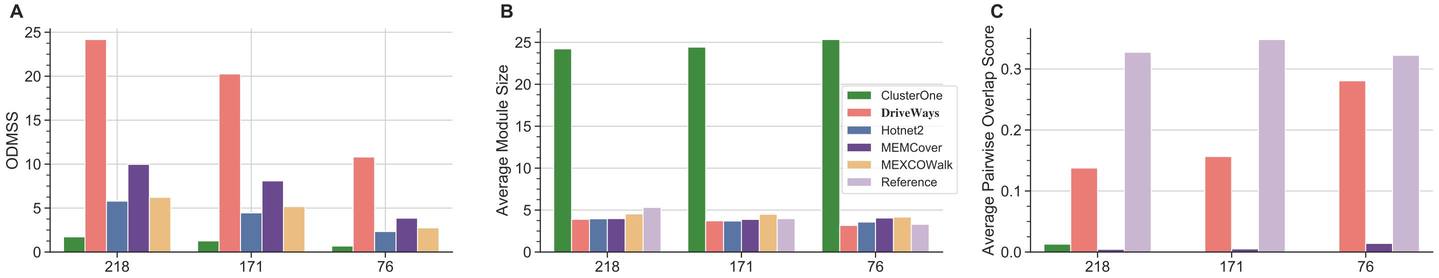

Figure S11: A)Overlapping driver module set score (ODMSS) obtained from lung cancer samples when δ_s parameter is determined based on the total size of KEGG_{CMlung}, Reactome_{CMlung} and BioCarta_{CMlung} pathways, respectively. B) Average module sizes in the outputs of the methods under consideration for the shown δ_s values obtained from lung cancer samples. C) Corresponding average pairwise overlap scores.

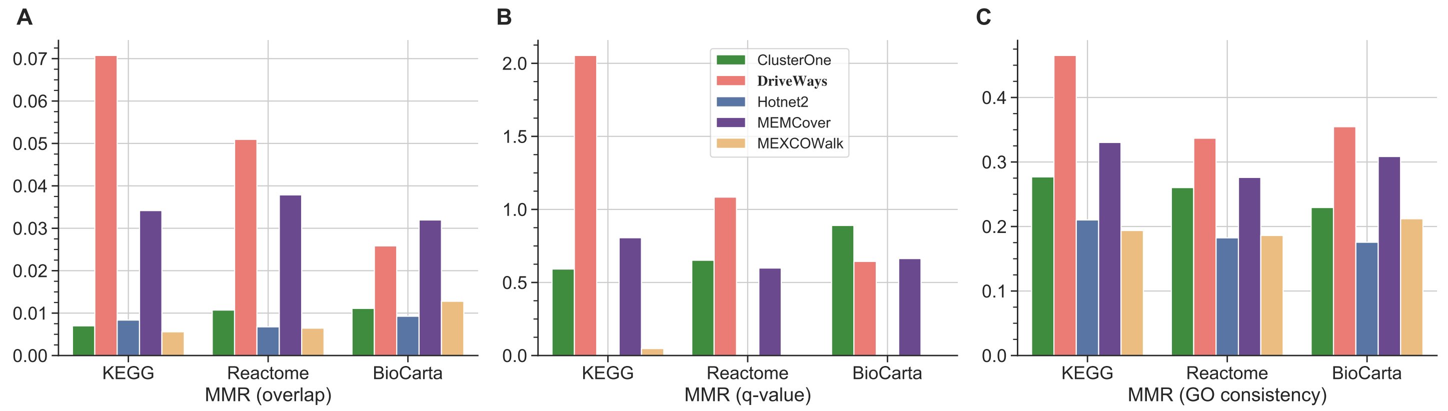

Figure S12: MMR scores of all methods calculated with three similarity metrics: A) Overlap score B) Hypergeometric test q-values C) GO consistency. The set of reference pathways at the x-coordinate of each plot correspond to KEGG_{CMlung}, Reactome_{CMlung}, and Biocarta_{CMlung}, from left to right.

1.4 MMR Examples

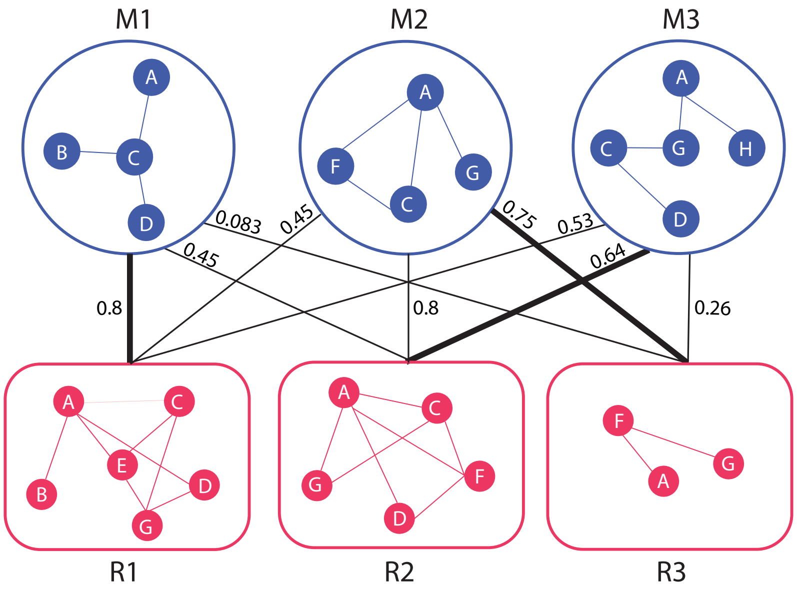

Figure S13: A toy example illustrating the calculation of Maximum Matching Ratio (MMR). M1,M2 and M3 are the predicted modules, and R1, R2 and R3 are the reference pathways. Edge weights correspond to the overlap score between the adjacent predicted module and reference pathway. Bold edges represent the edges in the maximum matching with maximum cardinality. MMR is (0.8 + 0.64 + 0.75)/3 = 0.73

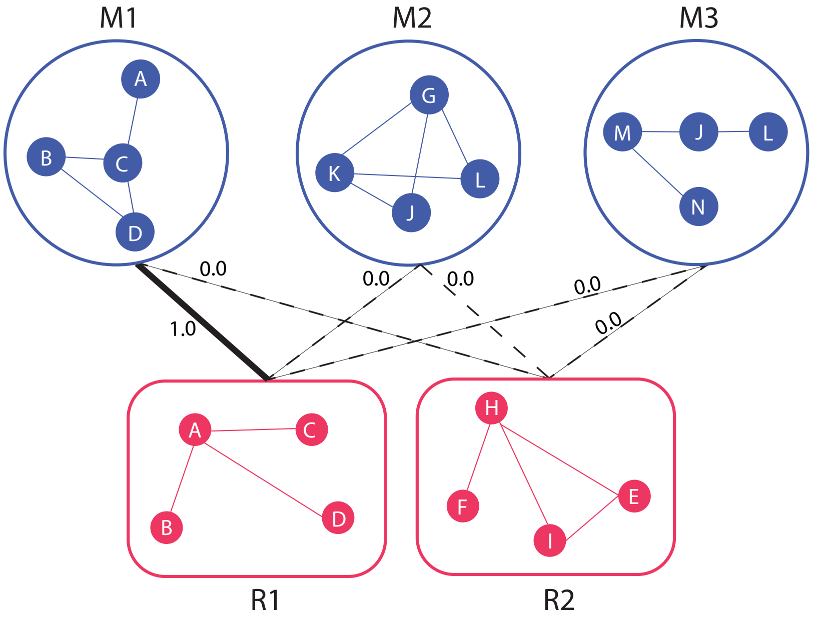

Figure S14: A toy example illustrating the effect of including zero weighted edges in the bipartite graph. If the zero edges were not present MMR would be 1, but considering those edges MMR is 1/3.

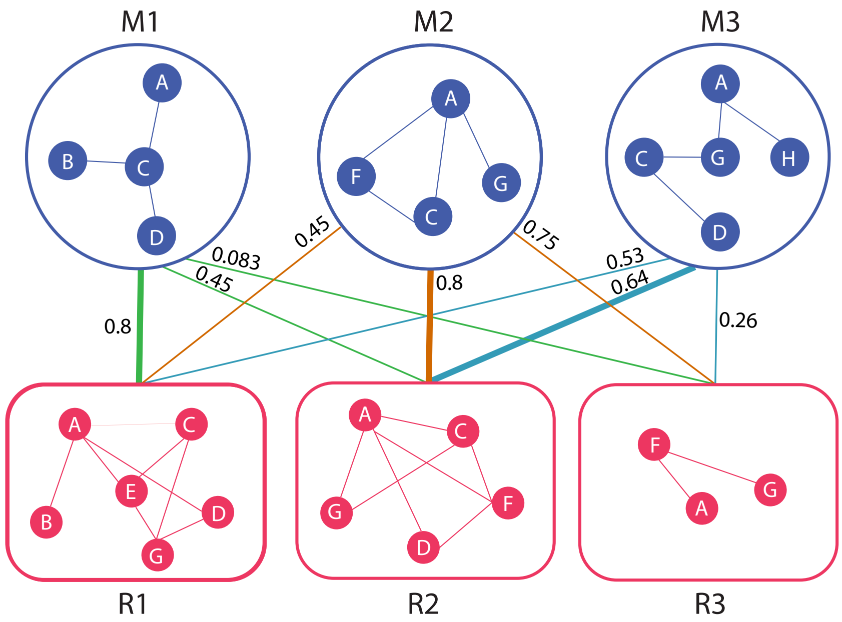

Figure S15: Illustration of finding the best match for each predicted module to evaluate precision. For each predicted module M_i, the best match is the maximum weighted edge between M_i and R_j such that $\exists j \in (1, 2, ..m)$ where m is the number of reference pathways.

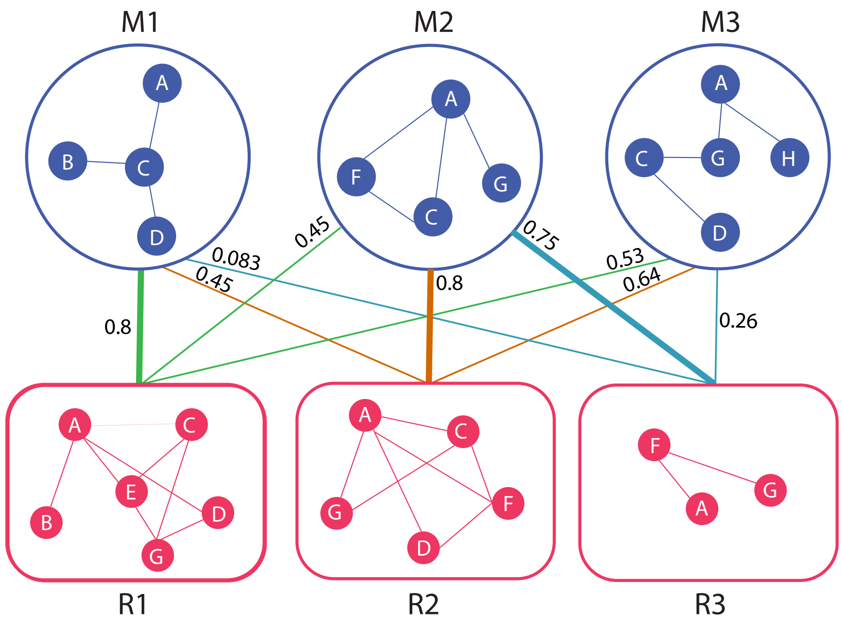

Figure S16: Illustration of finding the best match for each reference pathway to evaluate recall. For each reference pathway R_j, the best match is the maximum weighted edge between R_j and all M_i such that $\exists i \in (1, 2, ..n)$ where n is the number of predicted modules.

1.5 Robustness analysis on pan-cancer samples

We investigate the robustness of DriveWays results with respect to the δ_m parameter, the set of patients and the employed PPI network. DriveWays_δ_m4 and DriveWays_δ_m5 correspond to modified versions of DriveWays where δ_m is changed to 4 and 5, respectively. DriveWays_Bootstrap shows the results of running DriveWays 100 times on bootstrapped samples. DriveWays _HINT corresponds to a modified version of DriveWays where HINT network is employed as the input PPI. Below we show the evaluation results for these modified versions.

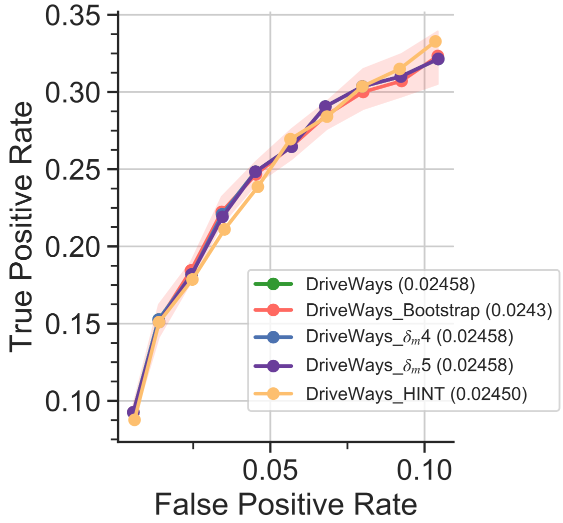

Figure S17: ROC curves calculated for $unique\_genes = 100, 200, ..., 1000$ from the outputs of different versions of DriveWays when pancancer samples are used as input and CGC is used for reference. The shaded region corresponds to the ROC curves of DriveWays when executed on 100 bootstrapped samples.

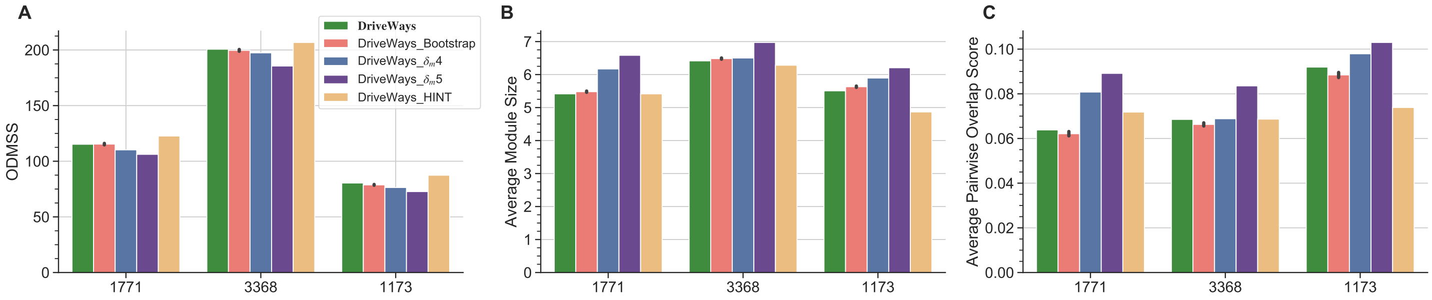

Figure S18: Statistics of output modules of different versions of DriveWays A) Overlapping driver module set score (ODMSS) obtained from pancancer samples when δ_s parameter is determined based on the total size of KEGG$_{CGC}$, Reactome$_{CGC}$ and BioCarta$_{CGC}$ pathways, respectively. B) Average module sizes in the outputs of the methods under consideration for the shown δ_s values obtained from pancancer samples. C) Corresponding average pairwise overlap scores.

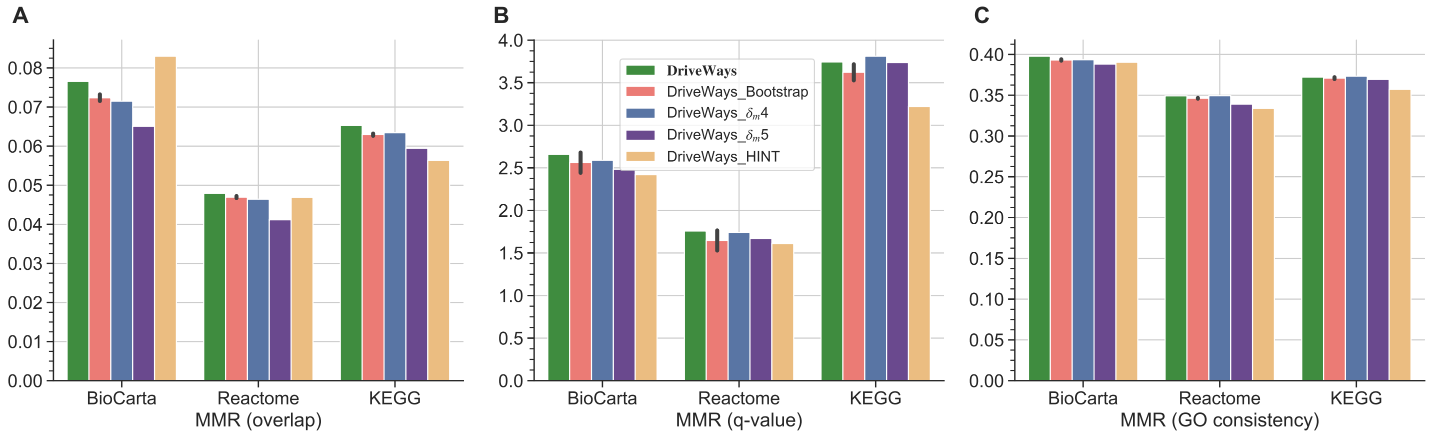

Figure S19: MMR scores of different versions of DriveWays with three similarity metrics: A) Overlap score B) Hypergeometric test q-values C) GO consistency. The set of reference pathways at the x-coordinate of each plot correspond to $KEGG_{CGC}$, $Reactome_{CGC}$, and $Biocarta_{CGC}$, from left to right.

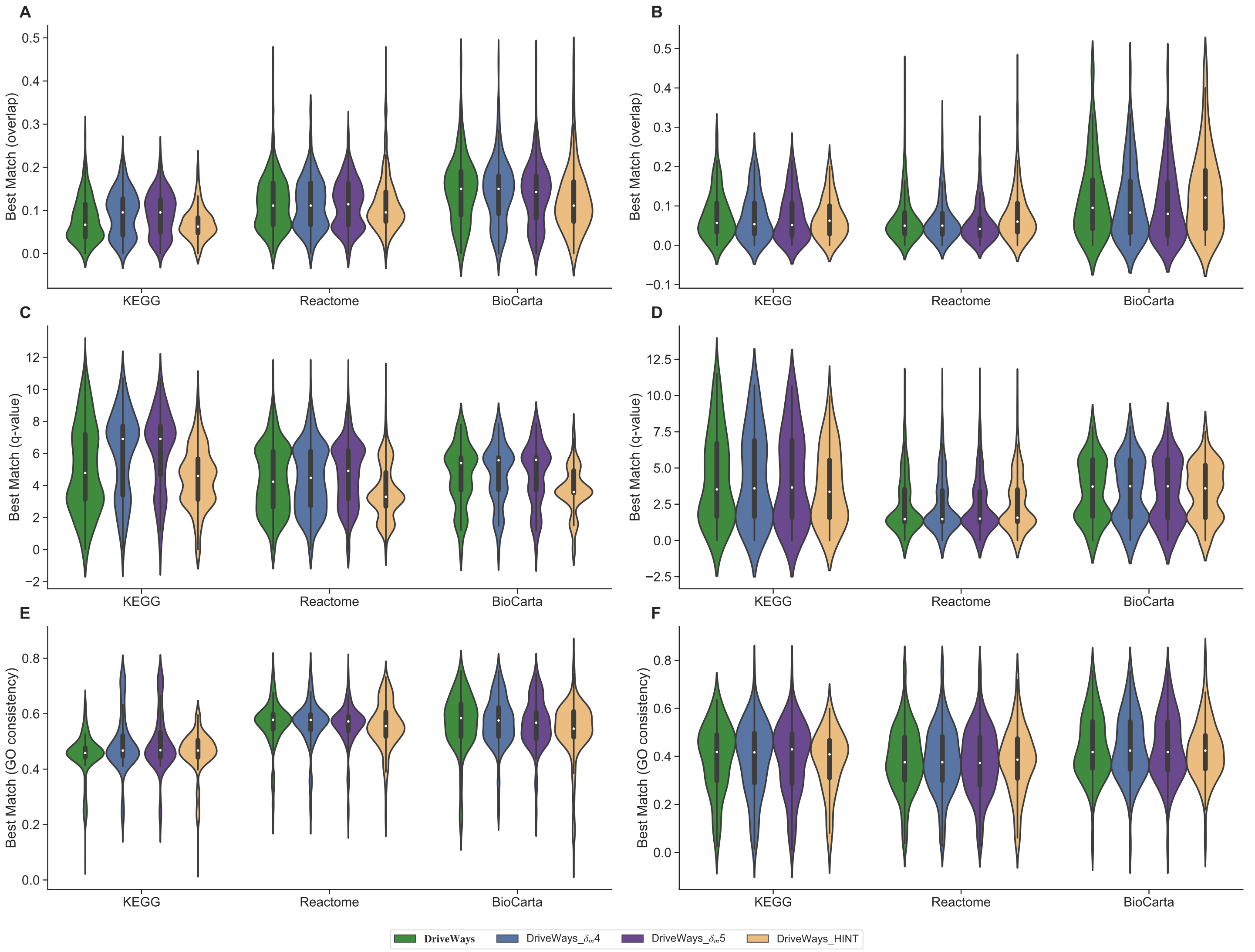

Figure S20: Evaluation of precision of different versions of DriveWays by comparing distribution of best match scores for each predicted module when pancancer samples are used as input; scores are calculated with (A) overlap score (C) q-value and (E) GO consistency. Evaluation of recall of different versions of DriveWays by comparing distribution of best match scores for each reference pathway when pancancer samples are used as input; scores are calculated with (B) overlap score (D) q-value (F) GO consistency.

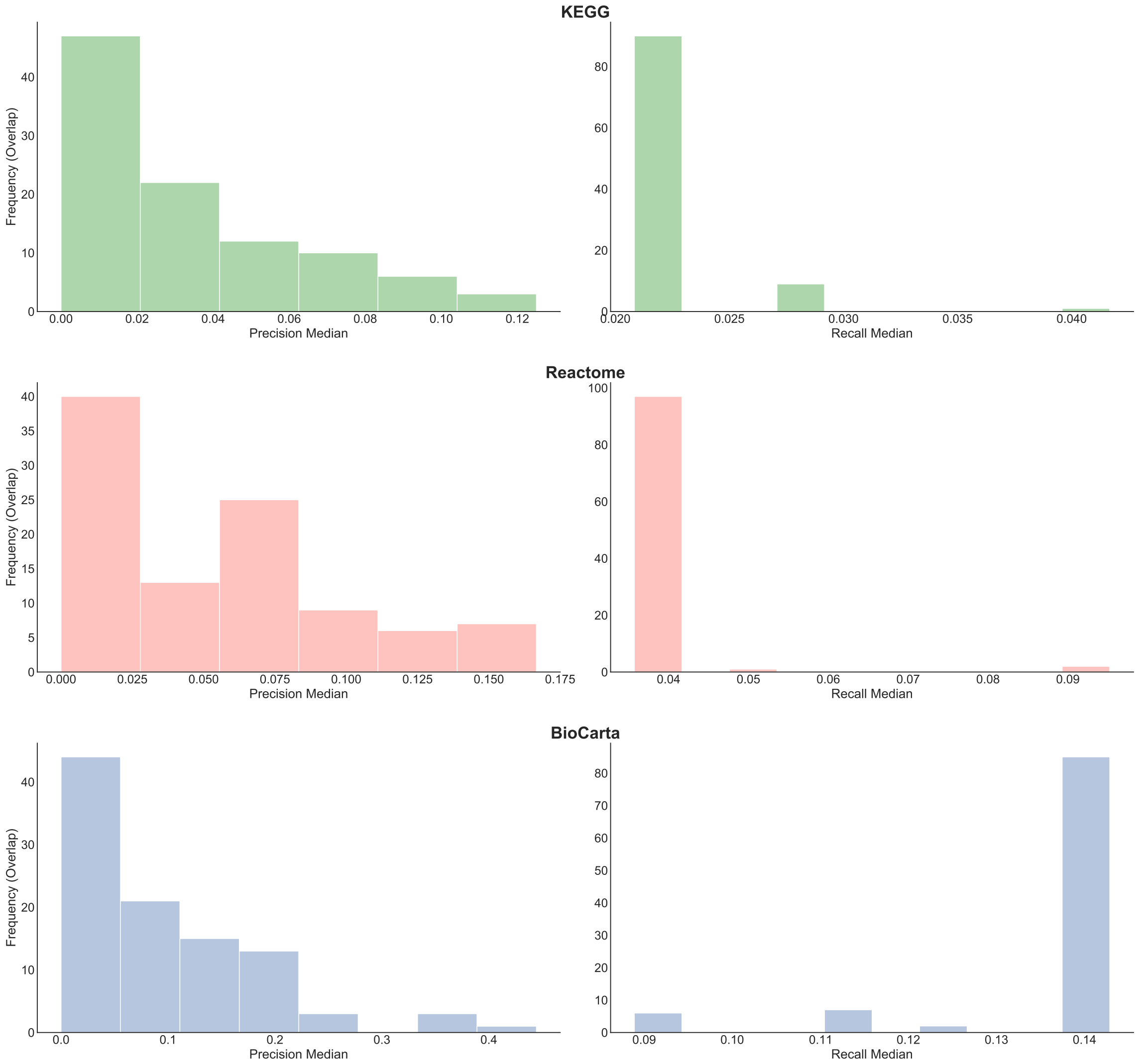

Figure S21: Histogram of median values computed across the best match scores of modules or reference pathways for each of the 100 boostrap runs. Match scores are calculated based on overlap score definition. The set of reference pathways of each plot correspond to KEGG_{CGC}, Reactome_{CGC}, and Biocarta_{CGC}, from top to bottom.

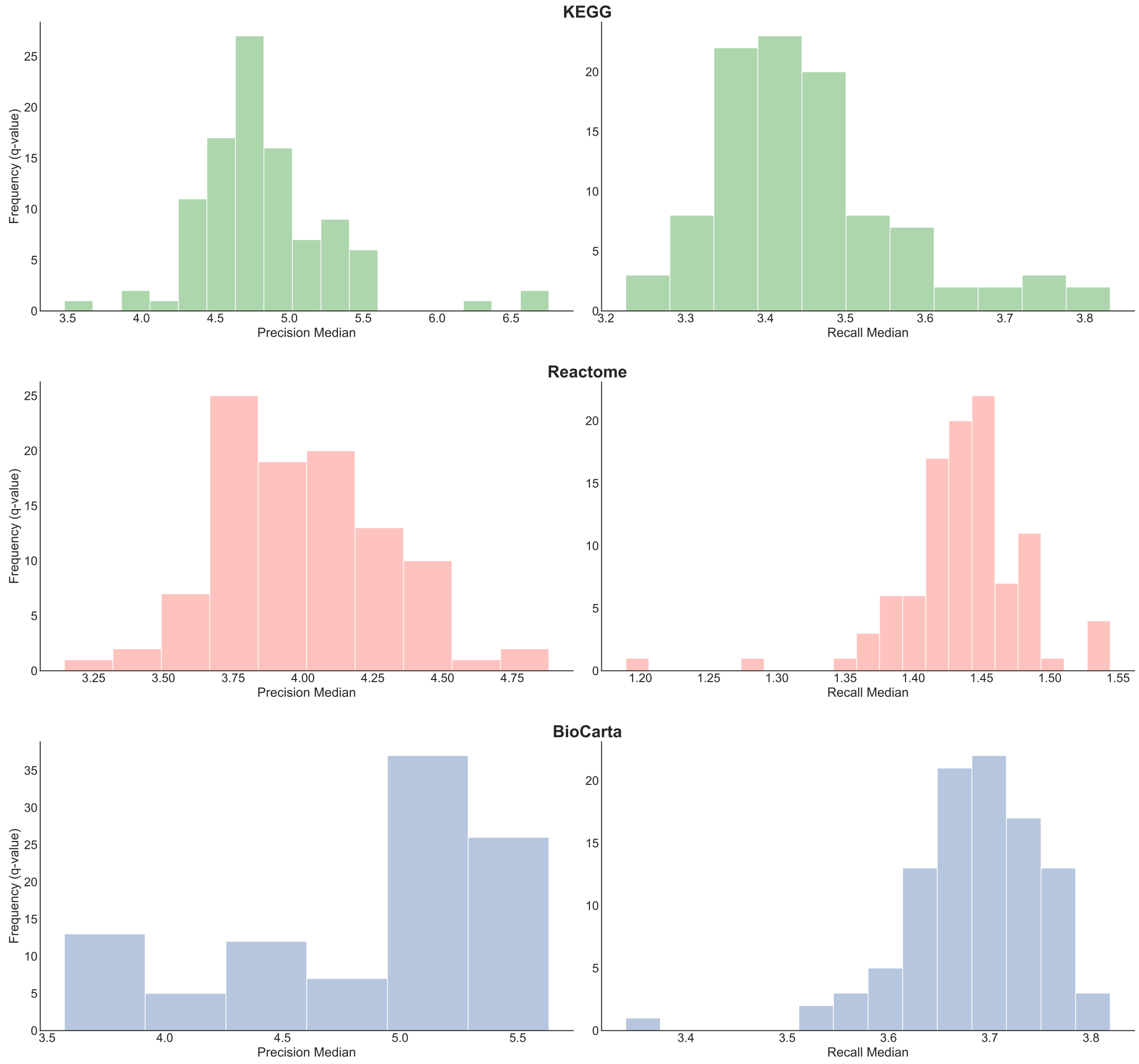

Figure S22: Histogram of median values computed across the best match scores of modules or reference pathways for each of the 100 boostrap runs. Match scores are calculated based on q-value score definition. The set of reference pathways of each plot correspond to KEGG_{CGC}, Reactome_{CGC}, and Biocarta_{CGC}, from top to bottom.

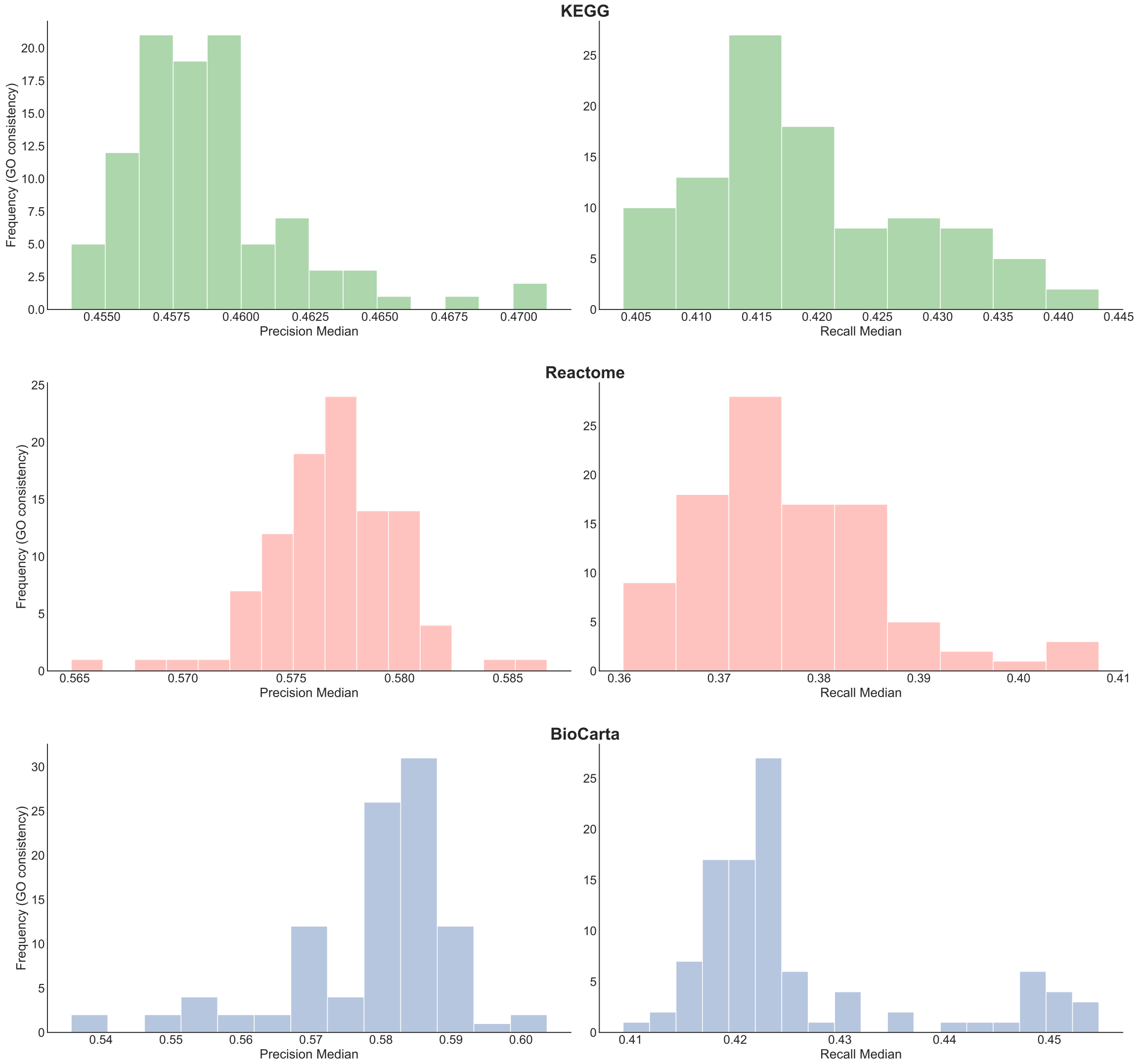

Figure S23: Histogram of median values computed across the best match scores of modules or reference pathways for each of the 100 boostrap runs. Match scores are calculated based on GO consistency score definition. The set of reference pathways of each plot correspond to KEGG_{CGC}, Reactome_{CGC}, and Biocarta_{CGC}, from top to bottom.

Table S1: t and d values selected by BayesOpt procedure

Reference	Input Data	t	d
KEGG_{CGC}		1.043	2.435
Reactome_{CGC}	Pan-cancer	1.0	3.0
BioCarta_{CGC}		1.017	3.011
$\text{KEGG}_{CMbreast}$		1.080	5.0
$\text{Reactome}_{CMbreast}$	Breast cancer	0.926	5.0
$\text{BioCarta}_{CMbreast}$		0.986	3.126
KEGG_{CMlung}		1.086	2.0
Reactome_{CMlung}	Lung cancer	1.096	5.0
BioCarta_{CMlung}		1.088	5.0

Table S2: Statistics of reference pathway databases

Reference	# of total genes	# of pathways	# of unique genes
KEGG_{CGC}	1771	104	330
Reactome_{CGC}	3368	337	360
BioCarta_{CGC}	1173	149	188

Table S3: Statistics on predicted modules of considered methods

Reference		ClusterOne	DriveWays	Hotnet2	MEMCover	MEXCOWalk
KEGG_{CGC}	# of modules	118	327	40	395	216
	# of unique genes	1291	571	1770	1386	1770
Reactome_{CGC}	# of modules	261	525	13	727	350
	# of unique genes	2212	909	3368	2649	3368
BioCarta_{CGC}	# of modules	73	213	57	262	141
	# of unique genes	893	326	1173	921	1173

Table S4: Pan-cancer average F1 scores

Reference		ClusterOne	**DriveWays**	Hotnet2	MEMCover	MEXCOWalk
	overlap	0.018	**0.078**	0.003	0.031	0.014
KEGG_{CGC}	q-value	1.412	**4.589**	0.499	0.856	0.301
	GO consistency	0.329	**0.419**	0.196	0.333	0.304
	overlap	0.025	**0.084**	0.001	0.042	0.019
Reactome_{CGC}	q-value	0.915	**3.125**	0.156	0.439	0.108
	GO consistency	0.351	**0.458**	0.179	0.378	0.326
	overlap	0.019	**0.132**	0.004	0.048	0.018
BioCarta_{CGC}	q-value	1.089	**3.91**	0.035	0.917	0.134
	GO consistency	0.341	**0.495**	0.200	0.374	0.307

Table S5: BRCA average F1 scores

Reference		ClusterOne	**DriveWays**	Hotnet2	MEMCover	MEXCOWalk
$\text{KEGG}_{CMbreast}$	overlap	0.015	**0.086**	0.001	0.037	0.008
	q-value	0.633	**2.848**	0.231	0.676	0.382
	GO consistency	0.318	**0.455**	0.195	0.341	0.265
$\text{Reactome}_{CMbreast}$	overlap	0.019	**0.070**	0.001	0.031	0.014
	q-value	0.491	**2.417**	0.060	0.273	0.116
	GO consistency	0.346	**0.463**	0.203	0.350	0.296
$\text{BioCarta}_{CMbreast}$	overlap	0.015	**0.097**	0.001	0.044	0.006
	q-value	0.434	**2.396**	0.086	0.696	0.350
	GO consistency	0.342	**0.463**	0.183	0.352	0.236

Table S6: LUAD average F1 scores

Reference		ClusterOne	**DriveWays**	Hotnet2	MEMCover	MEXCOWalk
$\text{KEGG}_{CMbreast}$	overlap	0.009	**0.129**	0.011	0.048	0.008
	q-value	0.902	**2.948**	0.131	0.929	0.166
	GO consistency	0.285	**0.589**	0.276	0.373	0.246
$\text{Reactome}_{CMbreast}$	overlap	0.011	**0.115**	0.012	0.063	0.011
	q-value	0.770	**2.114**	0.163	0.956	0.231
	GO consistency	0.264	**0.547**	0.241	0.371	0.246
$\text{BioCarta}_{CMbreast}$	overlap	0.010	**0.066**	0.010	0.044	0.015
	q-value	0.985	**1.453**	0.072	0.898	0.163
	GO consistency	0.244	**0.546**	0.214	0.374	0.271
